# In-depth insight into tumor-infiltrating stromal cells linked to tertiary lymphoid structures and their prospective function in cancer immunotherapy

**DOI:** 10.1186/s40164-025-00695-8

**Published:** 2025-08-10

**Authors:** Maedeh Radandish, Niloofar Mashhadi, Amir Hossein Aghayan, Motahareh Taghizadeh, Sara Salehianfard, Sheida Yahyazadeh, Omid Vakili, Somayeh Igder

**Affiliations:** 1 Department of Immunology, School of Medicine, Isfahan University of Medical Sciences, Isfahan, Iran; 2 Department of Immunology, School of Medicine, Shiraz University of Medical Sciences, Shiraz, Iran; 3 Autoimmune Diseases Research Center, Shiraz University of Medical Sciences, Shiraz, Iran; 4 Department of Medical Laboratory Sciences, School of Paramedical Sciences, Zanjan University of Medical Sciences, Zanjan, Iran; 5 Department of Clinical Biochemistry, School of Medicine, Shiraz University of Medical Sciences, Shiraz, Iran; 6 Department of Clinical Biochemistry, School of Medicine, Shahid Sadoughi University of Medical Sciences, Yazd, Iran; 7 Department of Clinical Biochemistry, School of Pharmacy and Pharmaceutical Sciences, Isfahan University of Medical Sciences, Isfahan, Iran; 8 Department of Clinical Biochemistry, School of Medicine, Ahvaz Jundishapur University of Medical Sciences, Ahvaz, Iran

**Keywords:** Neoplasms, Cancer immunotherapy, Tumor microenvironment, Stromal cell immunomodulation, Tertiary lymphoid structures, Tumor infiltration

## Abstract

**Background and purpose:**

The tumor microenvironment (TME) is widely acknowledged as a pivotal regulator of cancer progression. However, the dualistic role of tertiary lymphoid structures (TLSs), which serve as critical immune hubs within the TME, remains incompletely characterized, particularly with respect to their context-dependent capacity to either inhibit or facilitate tumor development. This review aims to synthesize current understanding of the complex interactions between stromal cells and TLSs, addressing existing gaps in mechanistic insight and exploring therapeutic avenues to exploit TLS plasticity.

**Key reviewed topics:**

The current study critically reviews the mechanisms by which stromal components, including cancer-associated fibroblasts and endothelial cells, contribute to TLS neogenesis through chemokine-mediated recruitment of lymphocytes. Furthermore, it highlights the dual functional roles of TLSs as sites of both anti-tumor immune activation and immunosuppression, notably via the enrichment of regulatory T cells. The clinical implications of mature TLS presence, particularly their association with improved patient prognosis and enhanced therapeutic responsiveness, are also analyzed.

**Main conclusions:**

TLSs demonstrate a bifunctional nature, wherein their spatial organization and dynamic interactions with stromal elements dictate the balance between immune activation and tolerance within the TME. While mature TLSs are generally correlated with favorable clinical outcomes, their potential to foster immunosuppressive microenvironments necessitates the development of precision-targeted interventions. The interplay between stromal cells and TLSs represents a promising therapeutic axis for modulating the tumor immune milieu.

**Future perspectives:**

Future research should prioritize strategies aimed at promoting TLS maturation, disrupting immunosuppressive niches, and integrating TLS-modulating agents with existing immunotherapeutic regimens to enhance clinical efficacy. Additionally, the identification of robust biomarkers reflective of TLS functional states and the rigorous validation of stromal-targeted therapies within combinatorial treatment frameworks are imperative for advancing translational applications.

**Graphical abstract:**

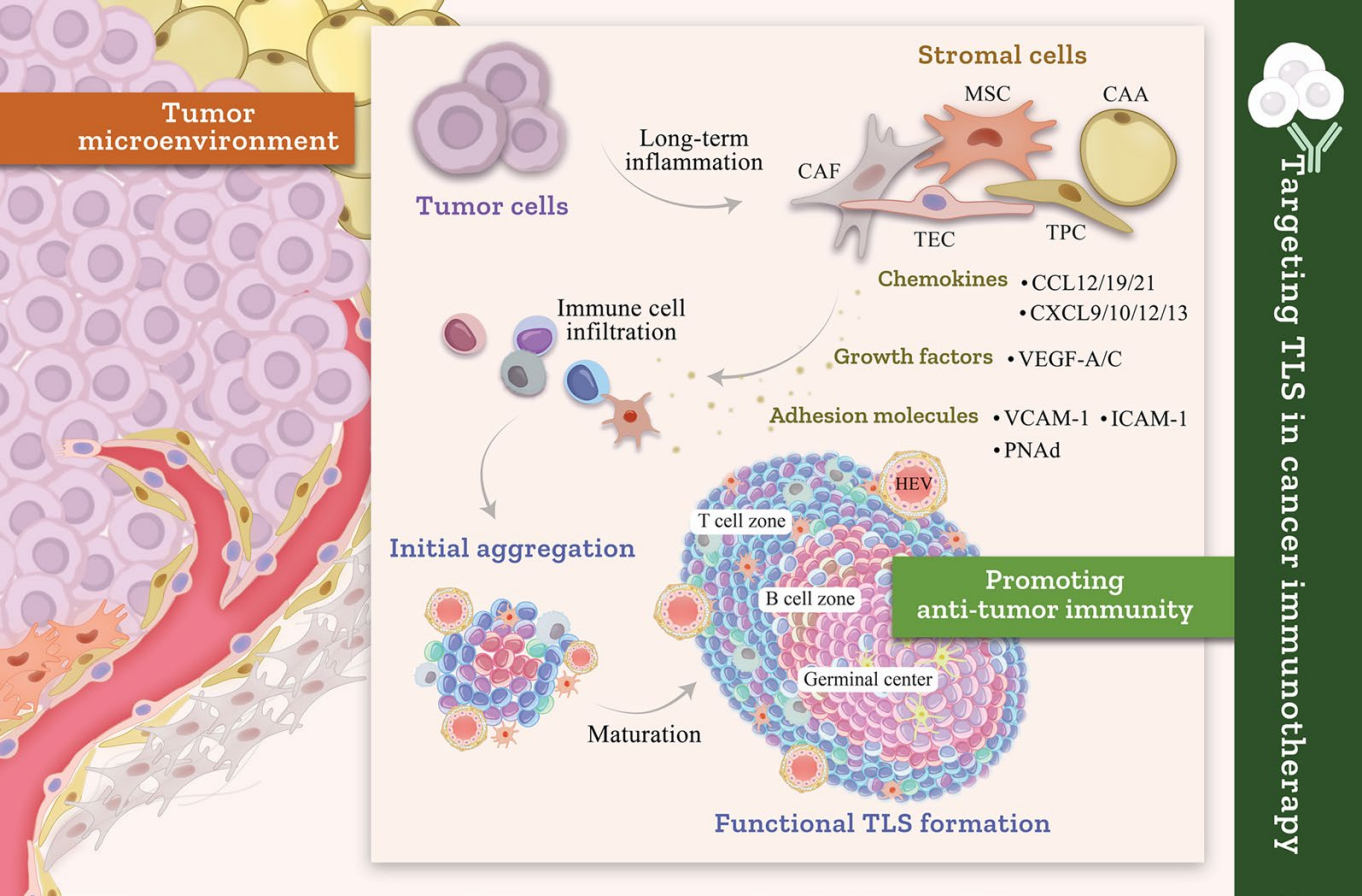

## Background

**Fig. 1:**
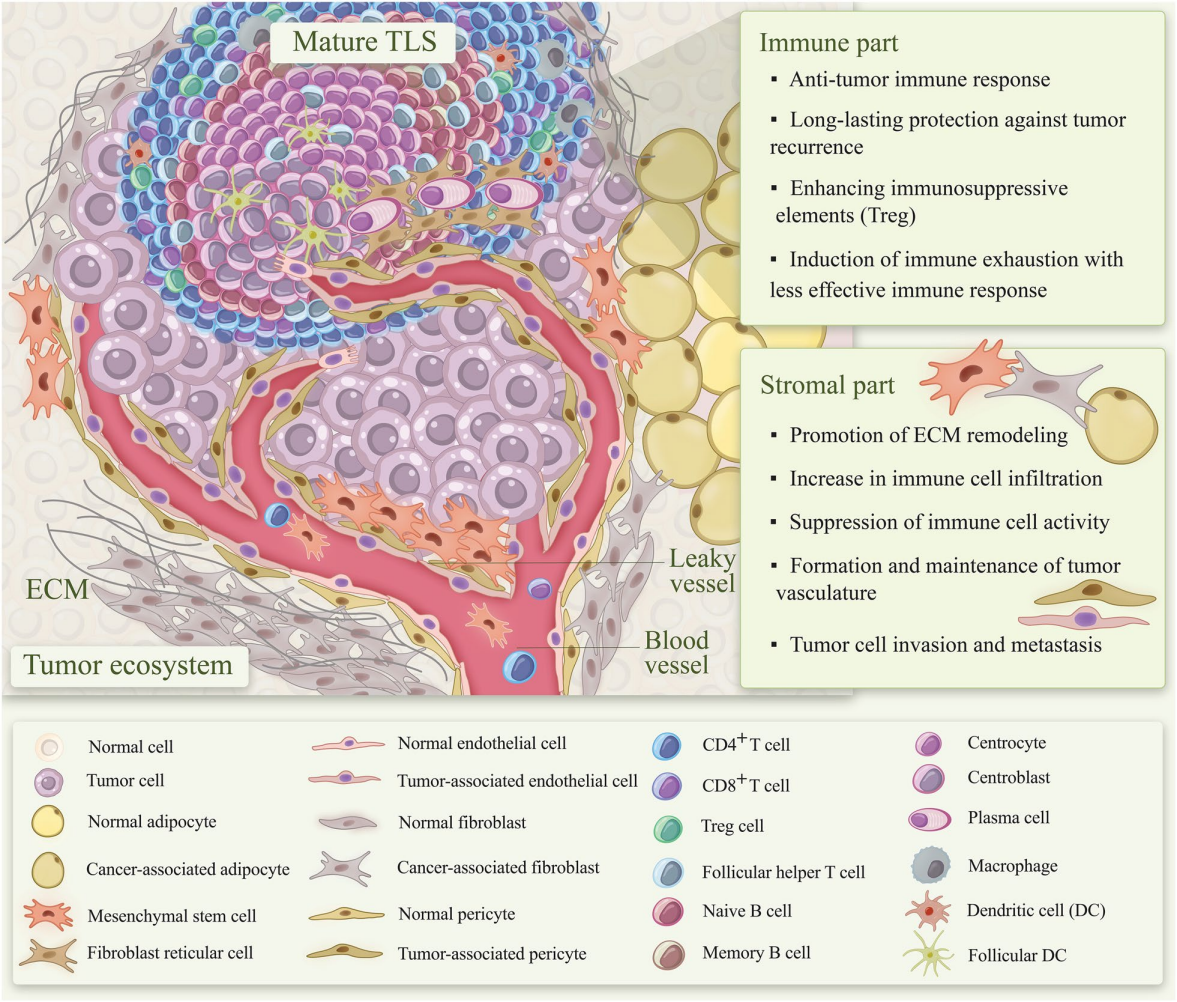
The tumor microenvironment and cancer: a schematic representation

Cancers originate from dysregulated cellular proliferation driven by a multifaceted interplay among genetic mutations, environmental influences, and the tumor microenvironment (TME) [1, 2] (Fig. 1). The TME, which comprises tumor cells and stromal components (incl. fibroblasts, endothelial cells, pericytes, immune cells, and extracellular matrix (ECM)), plays a crucial role in tumor growth, metastasis, immune evasion, and therapy response [3, 4]. Among the key features of TME are the development of tertiary lymphoid structures (TLSs) that resemble ectopic lymphoid tissue-like aggregates [5]. These structures develop in response to chronic inflammation, infection, or persistent antigenic stimulation within tumors [6]. TLSs form adaptively and locally in response to pathological stimuli [7]. TLSs function as sites for antigen presentation and lymphocyte activation, shaping tumor-specific immunity [8]. However, the role of TLS in cancer is complex and dual in nature. On one hand, TLSs can enhance immune surveillance and promote anti-tumor immunity [8, 9], while on the other hand, they may exert tumor-promoting effects. Stromal cells, including cancer-associated fibroblasts (CAFs), adipocytes (CAAs), endothelial cells, pericytes, and mesenchymal stem cells (MSCs), each play distinct roles within the tumor ecosystem [10]. Stromal cells modulate immune responses in the TME and affect the recruitment, activation, and function of immune cells [10, 11]. For instance, CAFs promote ECM remodeling, the migratory phenotype of tumor cells, as well as immune cell infiltration [12, 13]. MSCs take part in immune modulation by transducing signals that either downregulate or upregulate the immune response [14, 15]. Conversely, endothelial cells and pericytes are vital for the formation and maintenance of tumor vasculature and tumor growth [16–18]. TLSs are not static structures but evolve over time under the influence of innate and adaptive immune systems [19, 20]. One of the most important areas of interest in cancer immunology is the role of stromal cells in the formation and function of TLSs [21]. Regarding the TLS structure, both CAFs and endothelial cells are involved in the production of certain chemokines essential for the recruitment of lymphocytes, including C-X-C motif chemokine ligand 13 (CXCL13), C–C motif chemokine ligand 21 (CCL21), and CXCL12 [22]. Once immune cells are activated inside the TLS, they become organized and deployed at these sites. During this process, they interact with antigen-presenting cells (APCs) and other stromal components [23, 24]. Mature TLSs, when well-organized with distinct B and T cell zones, may serve as key sites for the initiation of robust anti-tumor immune response [19, 25]. Moreover, TLSs can function as sites of immune memory development thus offering long-lasting protection against tumor recurrence [9]. However, TLS formation can also have negative effects, such as providing a niche for regulatory T cells that suppress anti-tumor immunity and promote tumor progression [26]. Furthermore, the establishment of TLSs may lead to chronic inflammation, eventually resulting in immune exhaustion with less effective immune responses [6]. Understanding these complex dynamics is crucial for developing targeted therapies that can modulate TLSs’ formation and function to enhance anti-tumor immunity while minimizing immune suppression [27]. The interplay between stromal cells, TLSs, and tumor progression has been found to have great implications for cancer immunotherapy, opening new windows for improvement of therapeutic outcomes [28, 29]. Recent advancements in cancer immunotherapy, notably the development of immune checkpoint inhibitors (ICIs) and adoptive cell therapies (ACTs), including chimeric antigen receptor T-cell (CAR-T) therapy, have underscored the pivotal role of the TME in modulating therapeutic efficacy and clinical outcomes [30, 31]. In this context, TLSs have emerged as a highly promising target for various immunotherapeutic strategies [32, 33]. Specifically, modulation of stromal cells to promote TLS formation or enhance their functional capacity may potentiate T-cell activation, thereby augmenting anti-tumor immune responses [8]. However, the capacity of TLSs to harbor immunosuppressive elements, including regulatory T cells (Tregs) and myeloid-derived suppressor cells (MDSCs), necessitates careful consideration of the microenvironmental context in which TLS-targeted interventions are applied [34, 35]. Strategies aimed at mitigating immunosuppressive influences within TLSs, such as depletion of Tregs or enhancement of dendritic cell function, hold potential to overcome immune evasion mechanisms and improve the efficacy of cancer immunotherapy [36].

Despite considerable progress in elucidating the tumor microenvironment and the involvement of TLSs in cancer, significant knowledge gaps remain. In particular, the precise mechanisms by which distinct stromal cell populations coordinate the formation and functional maturation of TLSs have yet to be fully characterized. Furthermore, the determinants that influence whether TLSs exert pro-tumorigenic or anti-tumorigenic effects across various cancer types require further investigation. The current review aims to address these deficiencies by providing a comprehensive synthesis of current research on the interplay between tumor-infiltrating stromal cells and TLSs. Through this critical analysis, we seek to highlight key areas for future research and propose potential strategies to modulate stromal-TLS interactions in order to enhance anti-tumor immunity and optimize therapeutic efficacy. Furthermore, this review centers on the role of tumor-infiltrating stromal cells in the genesis and function of TLSs and their attendant immunological implications in cancer therapy. We review key stromal cell subtypes, their bifunctional role in cancer immunity by mediating both anti-tumor responses and immune evasion, and the ontogeny and maturation of TLSs in modulating anti-cancer immunity. Additionally, we address the prognostic value of TLSs and the challenges inherent in their detection and quantitative assessment.

## Tumor-infiltrating stroma cell subtypes and functions

### Cancer-associated fibroblasts

Cancer-associated fibroblasts (CAFs) represent a complex and dynamic population of cells residing within the TME [10, 37]. In the context of malignancy, normal fibroblasts undergo phenotypic transformation into CAFs in response to diverse tumor-associated stimuli, including hypoxia, inflammation, oxidative stress, and growth factors such as transforming growth factor-beta (TGF-β), platelet-derived growth factor (PDGF), fibroblast growth factor (FGF), and epidermal growth factor (EGF) [38, 39]. Emerging evidence indicates that CAFs do not exclusively derive from resident fibroblasts; rather, they originate from multiple progenitor cell types contingent upon the tumor milieu. This cellular heterogeneity is exemplified by the differentiation of CAFs from sources including pancreatic stellate cells (PSCs), MSCs, adipocytes, and pericytes [40–42]. Such diversity in origin underpins the functional and phenotypic complexity observed among CAF populations across various malignancies.

Morphologically, CAFs resemble immature fibroblasts, characterized by large, spindle-shaped morphology and prominent nuclei [43]. Compared to their normal counterparts, CAFs exhibit altered signaling pathways, including upregulated TGF-β expression, dysregulated Wnt signaling, and modifications in Sonic hedgehog (Shh) pathway activity [10, 38, 43]. Activated CAFs express distinctive markers such as alpha-smooth muscle actin (α-SMA), fibroblast activation protein (FAP), and platelet-derived growth factor receptor (PDGFR), facilitating their discrimination from normal fibroblasts [38, 44]. While normal fibroblasts generally exert tumor-suppressive effects, CAFs predominantly promote tumor progression, although some subsets may exhibit inhibitory functions. For example, myofibroblastic CAFs (myCAFs), which localize proximal to tumor cells, secrete collagen and ECM components that confer a protective niche in pancreatic ductal adenocarcinoma [45]. Tumor-promoting CAF subsets, including inflammatory CAFs (iCAFs) and Zeb1-positive CAFs, play critical roles in facilitating tumor growth and metastasis [46].

CAFs contribute to tumorigenesis, invasion, and metastatic dissemination through multiple mechanisms [44]. In breast cancer, CAFs enhance the metastatic potential of premalignant and malignant epithelial cells, contrasting with normal fibroblasts that tend to maintain epithelial characteristics and inhibit metastasis [47]. This pro-metastatic effect is largely mediated by myCAFs, which secrete matrix metalloproteinases (MMPs) to degrade ECM and promote tumor cell migration. Additionally, myCAFs produce CXCL13, which may facilitate TLS formation by recruiting lymphocytes [48]. CAFs also induce malignant transformation of non-neoplastic cells via overexpression of factors such as estrogen, TGF-β, and hepatocyte growth factor (HGF) [49]. Zeb1-positive CAFs, in particular, promote tumor progression through these growth factors [50]. In lung cancer, CAFs enhance metastatic potential through activation of the IL-6/STAT3 signaling axis, with iCAFs identified as the principal subset driving cytokine-mediated metastasis [51]. Similarly, in gastric cancer, myCAFs promote tumor invasion by remodeling the ECM through secretion of matrix-modifying enzymes [52]. iCAFs contribute to immune evasion by releasing pro-inflammatory cytokines and directly influencing pancreatic cancer cells to facilitate tumor progression; their secretion of CXCL12 may also support TLS formation by lymphocyte recruitment [53].

CAFs play pivotal roles in mediating resistance to various therapeutic modalities; for instance, CAF-derived interleukins IL-6 and IL-8 increase resistance to cisplatin (CDDP) in head and neck squamous cell carcinoma and gastric cancer, with iCAFs being the predominant source of these cytokines [54, 55]. Exosomes containing miR-196a, secreted by CAFs, confer cisplatin resistance in advanced head and neck cancers by targeting CDKN1B, whereas neuregulin 1 (NRG1)-expressing CAFs contribute to trastuzumab resistance in HER2-positive breast cancer through activation of the HER3/AKT pathway; these exosomes primarily originate from myCAFs [56].

Given the critical involvement of CAFs in tumor progression and therapeutic resistance, multiple strategies aimed at counteracting their deleterious effects are under investigation [57]. These approaches include inhibition of CAF activation, depletion of CAF populations, and reprogramming of CAF phenotypes. For example, fibroblast activation protein (FAP)-targeting vaccines have demonstrated potential in breast cancer by eliciting immune responses that eliminate CAFs and attenuate tumor growth [12]. Additional strategies focus on modulating CAF activity via cytokine or signaling pathway interference, such as pirfenidone, which reduces immunosuppression in breast cancer, and calcipotriol, which inhibits CAF migration and proliferation [58–61]. Targeting tumor-promoting CAF subsets, such as G protein-coupled receptor 77 (GPR77)-expressing CAFs, has shown promise in enhancing therapeutic efficacy; combined administration of anti-GPR77 antibodies with chemotherapy improves tumor cell apoptosis and overall treatment response [62]. Studies targeting CAFs through markers including FAP and GPR77 are ongoing and have yielded encouraging preliminary results. These interventions predominantly focus on myCAFs and iCAFs, which are integral to tumor progression and TLS modulation, by targeting their FAP expression and cytokine-mediated pathways, respectively [63]. Nonetheless, further research is warranted to develop effective and clinically translatable therapies targeting CAFs.

### Mesenchymal stem cells

Mesenchymal stem cells (MSCs) are multipotent stem cells that are found in a variety of tissues, including bone marrow, adipose tissue, umbilical cord, and even dental pulp [64]. These cells are known for their remarkable ability to differentiate into a wide range of cell types, including osteoblasts, chondrocytes, adipocytes, and myocyte [65]. They are negative for hematopoietic markers such as CD45, CD34, and CD14, but they show positive expression of mesenchymal markers, including CD73, CD90, and CD105 [66]. Due to their inherent plasticity and ability to secrete a variety of bioactive molecules, MSCs have garnered significant interest in regenerative medicine and cancer therapy [67, 68]. One of the most notable characteristics of MSCs is their intrinsic capacity to migrate and home to sites of tissue injury, inflammation, and tumorigenesis [68, 69]. MSCs are primarily involved in tissue maintenance and repair under normal physiological conditions [70]. They contribute to tissue homeostasis by releasing growth factors and cytokines that regulate cell survival, differentiation, and proliferation. However, their role in the TME is more complex and context-dependent [71]. MSCs migrate to the TME through interactions between their surface chemokine receptors (e.g., CCR-1, CCR-2, CCR-3) and tumor-secreted cytokines and chemokines, including endothelial cell selectin, MMPs, IL-8, and VEGF [72, 73]. Once they reach the TME, MSCs can differentiate into CAFs or other stromal cell types, contributing to tumor growth and metastasis [74]. The dual role of MSCs as both potential therapeutic agents and tumor promoters has made them central to cancer research. They are involved in nearly all stages of cancer progression, supporting early tumor growth by promoting angiogenesis, fostering a cancer-friendly environment, and helping tumors evade immune detection [75]. MSCs promote tumor progression primarily by secreting pro-inflammatory cytokines and growth factors such as VEGF, IL-6, TNF-α, and SDF-1. These factors are essential for tumor cell proliferation, survival, and migration [76]. By secreting cytokines such as TGF-β and IL-10, MSCs can suppress the activity of immune cells, including natural killer (NK) cells, cytotoxic T lymphocytes (CTLs), and dendritic cells (DCs) in a variety of cancerous and autoimmune conditions [77–79]. This immunosuppressive environment allows tumor cells to evade immune detection and continue proliferating. Furthermore, MSCs can recruit regulatory T cells (Tregs) to the TME, particularly in animal models of systemic lupus erythematosus, which further dampens anti-tumor immune responses [79]. This creates a vicious cycle, continuously suppressing the immune system and fostering an environment that allows tumor cells to grow and metastasize.

A key factor in MSCs’ role in cancer progression is their ability to migrate toward tumors, driven by chemotactic signals, particularly the CXCR4/SDF-1 axis [80]. The CXCR4 receptor on MSCs interacts with Stromal Cell-Derived Factor 1 (SDF-1), a chemokine from tumor cells and surrounding stroma, guiding MSCs toward the tumor site to support tumorigenesis. In some cases, tumor cells express CXCR4, creating a feedback loop that enhances tumor growth. Additionally, molecules like Macrophage Migration Inhibitory Factor (MIF), which interact with receptors such as CXCR2 and CXCR4, also contribute to MSC migration to the TME [81, 82]. Moreover, hypoxia, a hallmark of the TME, plays a crucial role in enhancing MSC migration [83, 84]. Hypoxic conditions can stabilize HIF-1α, which increases the expression of chemokine receptors and growth factors such as SDF-1, boosting MSC motility and promoting their migration toward tumors [84]. This hypoxia-driven MSC recruitment is of great significance in solid tumors, such as glioblastoma and breast cancer, where hypoxic regions are commonly seen [84]. Although MSCs can promote tumor progression, their ability to home to tumor sites makes them promising candidates for cell-based cancer therapies, allowing targeted delivery of therapeutic agents to tumors while reducing systemic side effects [10]. One of the most notable applications of MSCs in cancer therapy is their use as delivery vehicles for oncolytic viruses (OVs), which are viruses that selectively infect and kill cancer cells [85, 86]. MSCs have been genetically engineered to carry and deliver OVs directly to tumors, taking advantage of their natural migratory properties. For example, in a clinical trial focused on osteosarcoma, MSCs were used to deliver oncolytic adenoviruses (OAds) to tumor sites [87]. The results demonstrated significant tumor regression and increased immune cell infiltration, indicating that MSC-based therapy can enhance the immune response against cancer. A phase I/II clinical trial (NCT01844661) conducted in 2020 further validated the safety and efficacy of MSC-delivered OAds in children and young adults with metastatic osteosarcoma, reporting partial responses in 22% of patients and stable disease in 44%, with no dose-limiting toxicities [88]. Additionally, MSCs can be modified to express suicide genes, like cytosine deaminase, which convert non-toxic compounds into cytotoxic drugs at the tumor site [89].

Furthermore, MSCs can be engineered to carry chemotherapeutic agents such as doxorubicin, paclitaxel, and gemcitabine directly to tumor cells [90]. MSCs as drug carriers targeting tumors, can improve drug efficacy and minimize hazards to healthy tissues. They also regulate the tumor immune microenvironment, just like the tissue resident macrophages do [91]. Although MSCs have immunosuppressive effects that promote tumor growth, they can be modified to boost immune responses against tumors [10]. For example, MSCs can be engineered to express immune-modulatory proteins such as IFN-α or IL-12, which have potent anti-tumor effects [92, 93]. Modified MSCs have shown promise in preclinical studies by improving the efficacy of CAR-T cell therapies in solid tumors and boosting the overall anti-tumor immune response [94]. MSCs have also been shown to promote tissue repair and regeneration, making them a valuable adjunct to traditional cancer therapies. For instance, MSCs can help alleviate graft-versus-host disease (GVHD) following hematopoietic stem cell transplantation (HSCT) by modulating immune responses and promoting tissue healing [95, 96]. Clinical trial for acute GVHD reported complete responses in 50% of patients and partial responses in 30% after MSC infusions [97]. Moreover, a systematic review of MSC clinical applications reported that umbilical cord-derived MSCs improved functional recovery in patients with chemotherapy-induced myelosuppression, with 60% of treated patients showing restored hematopoietic function within 3 months [98].

### Cancer-associated adipocytes

Cancer-associated adipocytes (CAAs) have emerged as pivotal players in the TME, significantly influencing tumor initiation, progression, and metastasis [99]. These adipocytes, once recruited to the TME, undergo a transformation that alters their normal function, turning them into pro-tumorigenic cells, such as in colorectal cancer [100]. CAAs contribute to tumor progression by secreting cytokines, adipokines, lipid metabolites, and exosomes, creating a pro-inflammatory, immunosuppressive, and nutrient-rich environment that supports tumor growth and spread. They also interact with stromal, immune, and tumor cells, shaping the TME to promote tumor survival, metastasis, and resistance to therapy [99]. Leptin, a key adipokine from CAAs, increases tumor cell invasion by upregulating invasion-related proteins like MMP-9 and S100A7 [101, 102]. In addition, in breast cancer cells, leptin contributes to the epithelial-mesenchymal transition (EMT), which is a crucial process that enables cancer cells to acquire migratory and invasive properties, promoting metastasis [103]. On the other hand, adiponectin, another adipokine, has protective effects in normal tissues but becomes pro-tumorigenic, such as in breast cancer, when secreted by CAAs [104]. It helps cancer cells upregulate the expression of CCL5, a chemokine that promotes the migration and invasion of triple negative breast cancer cells [105]. Besides adipokines, CAAs secrete pro-inflammatory cytokines like IL-6 and IL-8; IL-6 activates the JAK/STAT3 signaling pathway in tumor cells, promoting their growth and metastasis [106]. Moreover, IL-8, produced by CAAs, promotes angiogenesis, aiding in the development of a vascular network that supports tumor growth and allows for metastasis to distant organs, particularly in breast cancer [107]. The breakdown of fat within CAAs leads to the release of FFAs, which are readily taken up by cancer cells through specific receptors such as CD36 and Fatty Acid Transport Protein 1 (FATP1) [108]. These fatty acids are used by tumor cells in various ways, including the synthesis of cell membranes, energy production through β-oxidation, and lipid-derived signaling [108, 109]. For example, FFAs have been shown to activate the NF-κB signaling pathway, which enhances inflammation, promoting cancer cell proliferation and survival [110]. In prostate cancer, FABP4 aids cancer cell growth by supplying lipids needed for rapid division [111]. CAAs also regulate the immune microenvironment by releasing exosomes with miRNAs, which affect immune cells like macrophages [112]. Macrophages recruited by CAAs are polarized into the pro-tumor M2 phenotype, promoting tumor progression by enhancing angiogenesis, immune suppression, and cancer cell migration. In breast cancer, miRNA-155 transfer via exosomes from CAAs increases tumor-associated macrophages (TAMs), aiding metastasis [113]. Visfatin, an adipokine secreted by CAAs, promotes M2 macrophage polarization and enhances glycolysis in tumor cells, helping tumors survive under low oxygen conditions, which is common in solid tumors like breast cancer [114]. In addition to secreting cytokines, adipokines, and lipid metabolites, CAAs contribute to tumor metastasis by transdifferentiating into cell types like CAFs and pericytes; these transformed adipocytes aid in angiogenesis and create a supportive tumor stroma that promotes cancer cell survival and metastasis, such as in gastric cancer [115]. Furthermore, CAAs can undergo a process of dedifferentiation, acquiring characteristics of CAFs, which support tumor progression by remodeling the extracellular matrix and facilitating cancer cell invasion [116]. Additionally, CAAs can differentiate into pericytes, which are essential for the formation and stabilization of blood vessels [99]. By participating in the construction of blood vessel walls, CAAs contribute to tumor vascularization, enabling the tumor to grow and metastasize [117]. The MSC-like plasticity of CAAs, particularly their trans-differentiation into CAFs or pericytes, offers a therapeutic target to disrupt tumor-supportive stromal remodeling [50]. Inspired by CAF-targeting strategies, inhibiting TGFβ signaling, which drives CAF plasticity and mesenchymal transformation, could suppress CAA trans-differentiation into CAFs, reducing desmoplastic stroma formation in cancers [118]. Similarly, targeting Hedgehog signaling, which reprograms CAFs to support cancer stemness, may prevent CAA transformation into tumor-supportive pericytes, limiting vascularization in tumors such as pancreatic cancer [119]. Bone marrow-derived adipocytes aid cancer cell recruitment to the bone, supporting bone metastasis. The dedifferentiation of CAFs into a stem-like state enhances their tumor-supportive functions and presents another therapeutic opportunity. Drawing from CAF strategies, inhibiting netrin-1, a CAF-derived factor that promotes cancer cell stemness, could target CAA-derived factors that sustain dedifferentiated, tumor-supportive states, thereby reducing their plasticity-driven effects on tumor progression [120, 121].

Targeting CAAs, with drugs like metformin, offers a promising strategy by reducing leptin secretion and reversing their dysfunctional phenotype, particularly in ovarian cancer [122]. Metformin can also inhibit tumor growth by normalizing adipocyte function and preventing the activation of pro-tumorigenic signaling pathways [122, 123]. Arctigenin can also suppress adipogenesis in preadipocytes and activated apoptosis in estrogen receptor (ER) positive breast cancer cells through modulating the expression of β-catenin [124].

### Tumor-associated endothelial cells

The formation of blood vessels within tumors, a process known as tumor angiogenesis, is one of the hallmarks of cancer [125]. This process sustains tumor growth and aids metastasis by providing a pathway for cancer cells to spread. Tumor-associated endothelial cells (TECs) are crucial for cancer development and progression [126]. TECs differ from normal endothelial cells (NECs) in structure, molecular traits, and metabolism, allowing them to promote tumor growth, metastasis, and resistance to treatment [127, 128]. Blood vessels formed by TECs are structurally and functionally abnormal, unlike the organized vessels in normal tissues. Tumor blood vessels are chaotic, irregularly shaped, and disorganized [129]. Tumor blood vessels are dilated, leaky, and poorly perfused, creating a hypoxic tumor microenvironment. The lack of proper endothelial cell junctions and incomplete perivascular coverage increases vascular permeability [130]. The abnormal blood vessels formed by TECs disrupt oxygen and nutrient delivery to the tumor, while allowing immune and tumor cells to enter the bloodstream, aiding metastasis [131]. VEGF and other pro-angiogenic factors like bFGF, PlGF, and angiopoietins (Angs) are overexpressed in TECs, which indicates the significant molecular differences between TECs and NECs [132]. These factors bind to receptors on TECs, triggering signaling pathways that promote endothelial cell proliferation, migration, and new blood vessel formation. For instance, VEGF activates its receptors (VEGFR-1 and VEGFR-2) on TECs, initiating a cascade of intracellular signals that enhance angiogenesis [133]. Other molecules, such as epidermal growth factor (EGF) and PDGF, further amplify the angiogenic response [134]. At the genetic level, TECs are also characterized by chromosomal instability, a common feature of cancer cells [135, 136]. TECs’ instability can lead to aneuploidy and chromosomal translocations, contributing to their aggressive phenotype. Studies show that TECs from metastatic tumors have more genetic abnormalities, higher proliferation rates, and increased resistance to chemotherapy compared to those from primary tumors [137]. These genetic abnormalities in TECs not only contribute to tumor progression but also complicate treatment strategies. Another significant difference between TECs and NECs is their metabolic profile [138]. TECs undergo metabolic reprogramming, relying on glycolysis for energy production (the Warburg effect), even in the presence of oxygen. This shift supports their high proliferative capacity, enabling tumor blood vessels to accommodate rapid tumor growth [138, 139]. In addition to glycolysis, fatty acid synthesis and serine biosynthesis pathways are also upregulated in TECs. For example, fatty acid synthase (FASN) is overexpressed in TECs, promoting lipid biosynthesis that is crucial for maintaining the structural integrity of the tumor vasculature [140]. The vascular network generated by TECs plays a key role in facilitating tumor metastasis [141]. TECs not only promote tumor growth and metastasis but also contribute to drug resistance, making tumors less responsive to chemotherapy and targeted therapies [142, 143]. For instance, TECs derived from particular cancers have been shown to exhibit resistance to the chemotherapeutic agent vincristine [144]. In this context, exosomes released by tumor cells can induce TECs to develop chemotherapy resistance; for instance, miRNA-1246 secreted by cancer cells promotes resistance to 5-FU in TECs by downregulating pro-apoptotic genes [145]. The TME plays a key role in modulating TEC behavior and contributing to therapy resistance. Targeting TECs has become a promising cancer treatment strategy. Anti-angiogenic therapies, such as monoclonal antibodies targeting VEGF (e.g., bevacizumab), aim to block TEC activity and disrupt tumor vasculature, and have been approved for clinical use in various cancers, particularly rectal cancer [146]. Bevacizumab works by binding to VEGF and preventing its interaction with VEGF receptors on TECs, thereby inhibiting endothelial cell proliferation and angiogenesis [146].

Recent studies, such as the IMpower150 study, have demonstrated enhanced efficacy when bevacizumab is combined with immune checkpoint inhibitors like atezolizumab, improving progression-free survival in lung cancer [147, 148]. In addition to monoclonal antibodies, small molecule tyrosine kinase inhibitors (TKIs), such as sorafenib, sunitinib, and pazopanib, have been developed to block VEGFR and other pro-angiogenic pathways [149].

Another promising approach involves targeting the Notch signaling pathway, which is crucial for endothelial cell differentiation and angiogenesis [150]. Notch1 is essential for the development of TECs, and inhibiting Notch signaling can lead to endothelial dysfunction, impaired angiogenesis, and reduced tumor growth [151]. Delta-like 4 (DLL4), a ligand for Notch, has been identified as a potential target for therapeutic intervention, and several DLL4 inhibitors are currently being investigated in human diseases [152]. Targeting the metabolic reprogramming of TECs is a promising therapeutic strategy. PPARα agonists like fenofibrate have shown potential in inhibiting TEC proliferation and angiogenesis by reducing VEGF production [153].

### Tumor-associated pericytes

Pericytes (PCs), also known as parietal cells, are mural cells that reside on the inner surface of blood vessels, particularly in microvessels, and play a critical role in the development and function of the vascular system [17]. These cells are essential for maintaining the integrity of blood vessels, regulating vascular permeability, and stabilizing the endothelial cell layer. PCs interact with endothelial cells to regulate angiogenesis, blood–brain barrier (BBB) function, and tissue homeostasis [17]. Importantly, tumor-associated pericytes (TPCs) not only contribute to these fundamental vascular functions but are also involved in tumor progression and metastasis through a variety of mechanisms [154, 155]. Pericytes are highly plastic cells that change phenotype based on signals from the microenvironment. In normal tissues, they support endothelial cells, stabilize blood vessels, and regulate permeability, but in the TME, their role becomes more complex [156]. TPCs exhibit functional alterations that support tumor growth, immune evasion, and metastasis [157]. TPCs are characterized by the expression of specific surface markers, including PDGFRβ, CD146 (MCAM), and NG2, which differentiate them from pericytes in normal tissues. While these molecules are also present on ECs and smooth muscle cells, they are upregulated in TPCs, playing key roles in tumor vasculature interactions [158]. During tumorigenesis, TPCs can be recruited or generated through mechanisms such as pericyte differentiation from fibroblasts or transdifferentiation of cancer cells in the TME [158, 159]. Furthermore, pericytes can be recruited by endothelial cells through signaling pathways activated by growth factors like PDGF, which triggers their involvement in tumor angiogenesis [160]. Reduced pericyte coverage allows cancer cells to escape into the bloodstream, promoting metastasis. Pericyte loss is also linked to increased vascular leakiness in glioblastomas, where dysfunctional blood vessels enhance tumor aggressiveness [161, 162]. Tumor blood vessels show structural and functional abnormalities, with pericytes playing a key role. Pericytes in tumors are more contractile than in normal tissues due to upregulation of glycolytic enzymes like hexokinase 2 (HK2) and signaling pathways such as Rho-associated coiled-coil containing protein kinase 2 (ROCK2) [163]. Pericytes can influence the recruitment and activation of immune cells, thus modulating the immune landscape of tumors. In particular, TPCs interact with various immune cell populations, including macrophages, dendritic cells, and T lymphocytes, to promote an immunosuppressive environment that supports tumor growth and metastasis [164, 165]. TPCs secrete factors like IL-33, CXCL12, and CXCL14, which recruit immune cells to promote a pro-tumor environment. Glioma-derived TPCs express high IL-33, attracting MDSCs to the tumor site [165]. MDSCs, in turn, inhibit the activity of cytotoxic T cells, promoting immune tolerance to the tumor. Additionally, pericytes in the TME can promote the polarization of macrophages toward an M2-like phenotype, which is associated with tumor-promoting functions such as immunosuppression, tissue remodeling, and angiogenesis [166]. TPCs play a dual role in tumor progression by facilitating growth and contributing to therapy resistance. They also mediate resistance to anti-angiogenic therapies like bevacizumab, which targets endothelial cells to inhibit new blood vessel formation in tumors [167]. However, these therapies often fail due to the presence of pericytes, which help stabilize the remaining tumor vasculature [168]. TPCs secrete pro-angiogenic factors and ECM components, supporting endothelial cell survival and making anti-angiogenic therapies less effective. Targeting pericytes alongside anti-angiogenic drugs could help overcome resistance and improve therapy outcomes. Given their key role in tumor progression, metastasis, and therapy resistance, strategies like inhibiting specific signaling pathways, glycolysis, and pericyte recruitment to tumor vessels offer potential therapeutic approaches. For example, Inhibiting the glycolytic activator PFKFB3 in tumor pericytes can normalize tumor vasculature and improve drug delivery [169]. Additionally, targeting PDGFRβ signaling in pericytes reduces their coverage on tumor blood vessels, increasing permeability and enhancing chemotherapy delivery [170]. Different subtypes of tumor-infiltrating stroma cells, including CAFs, MSCs, CAAs, TECs, and TPCs, along with their functions are summarized in Table 1.

**Table 1:** Different types of tumor-infiltrating stroma cells and their functions

Type of stromal cells in TME	Abbreviation	Sources of stromal cells	Stromal cells and tumors	Stromal cells and oncogenic mechanisms	Stromal cells and therapeutic resistance	Strategies for targeting stromal cells	References
Cancer-associated fibroblast	CAFs	Arisen from normal fibroblasts via tumor stimuli, incl. TGF-β^i^, PDGF^ii^, FGF^iii^, EGF^iv^ Subtypes: myCAFs, iCAFs, Zeb1 + CAFs, GPR77 + CAFs, PSCs, MSCs, adipocytes, pericytes	Promoting & progression tumor growth, invasion, and metastasis by Zeb1 + CAFs and GPR77 + CAFs Also, ECM^v^ remodeling and cytokines by myCAFs and iCAFs	Inducing immune evasion by iCAFs, malignant transformation by Zeb1 + CAFs, increased metastatic potential by myCAFs	Triggering resistance to therapies via cytokines (e.g., IL-6^v^^i^), exosomes, and signaling	–FAP^v^^ii^ vaccines for myCAFs –Cytokine modulation for iCAFs –Anti-GPR77 antibodies for GPR77 + CAFs	[12, 43, 44, 51, 59, 60]
Mesenchymal stem cells	MSCs	Arisen from multipotent mesenchymal stromal cells in bone marrow, adipose tissue, umbilical cord, and dental pulp; express CD73, CD90, CD105	Promoting tumor growth through chemokine-mediated migration (e.g., CXCR4^viii^/SDF-1^i^^x^), differentiation into CAFs, and modulation of the tumor microenvironment	Inducing angiogenesis, immune evasion (via TGF-β, IL-10^x^), and cytokine secretion (e.g., VEGF^x^^i^, IL-6, SDF-1	Driving resistance to therapies through immune suppression, Treg recruitment, and hypoxia-mediated mechanisms	–Engineered delivery of oncolytic viruses –Immune modulators (e.g., IFN-α^x^^ii^)	[64, 66, 68, 72, 82, 87, 92, 95, 96, 171]
Cancer-associated adipocytes	CAAs	Derived from mature adipocytes through TME signals, CAAs transition to a pro-tumorigenic state, driven by metabolic reprogramming and altered secretory profiles	Driving tumor progression and metastatic spread via cytokine secretion (e.g., IL-6, IL-8^x^^iii^), adipokine release (e.g., leptin), and lipid-derived signaling molecules	Driving tumor progression by enhancing cell invasion (via leptin), angiogenesis (via IL-8), immune suppression (via TGF-β, IL-10), and metabolic support through lipid provision	Driving resistance to therapies through lipid-mediated energy supply, secretion of pro-inflammatory cytokines, and establishment of an immunosuppressive niche	–Metformin to reduce leptin secretion –PPAR-γ^x^^iv^ agonists (e.g., rosiglitazone) to normalize adipocyte function –Blockade of exosome-mediated miRNA transfer to immune cells	[99, 100, 172]
Tumor-Associated Endothelial Cells	TECs	Arisen from normal endothelial cells	Promoting tumor growth and metastasis through the formation of abnormal, disorganized blood vessels that provide nutrients and facilitate cancer cell dissemination	Inducing tumor angiogenesis through secretion of pro-angiogenic factors (e.g., VEGF, bFGF^x^^v^), metabolic reprogramming (e.g., Warburg effect), and genetic instability, leading to aggressive tumor behavior	Triggering resistance to chemotherapy (e.g., vincristine, 5-FU^x^^vi^, doxorubicin) through genetic instability, activation of survival pathways, and exosome-mediated signaling	–Bevacizumab (anti-VEGF) –Sorafenib (TKI^x^^vii^) –DLL4 inhibitors (Notch pathway) –PPARα^x^^viii^ agonists (e.g., fenofibrate)	[151, 173]
Tumor-associated pericytes	TPCs	Arisen from mural cells on the inner surface of blood vessels, particularly in microvessels	Promoting tumor angiogenesis, vessel stabilization, and progression through abnormal blood vessel formation, enhancing metastasis and tumor aggressiveness	Inducing vascular dysfunction through increased permeability and disrupted endothelial-pericyte interactions, while modulating immune responses, recruiting immune cells, and promoting immune suppression	Triggering resistance to chemotherapy and anti-angiogenic therapies through vascular stabilization, enhanced drug clearance, and modulation of tumor microenvironment signaling	–PFKFB3^x^^ix^ inhibitors (glycolysis inhibition) –PDGFRβ^xx^ signaling inhibitors	[17, 155, 160, 174]

^i^Transforming Growth Factor-Beta

^ii^Platelet-Derived Growth Factor

^iii^Fibroblast Growth Factor

^iv^Epidermal Growth Factor

^v^Extracellular Matrix

^vi^Interleukin-6

^v^^ii^Fibroblast Activation Protein Vaccines

^v^^iii^C-X-C Chemokine Receptor Type 4

^ix^Stromal Cell-Derived Factor-1

^x^Interleukin-10

^xi^Vascular Endothelial Growth Factor

^x^^ii^Interferon-Alpha

^x^^iii^Interleukin-8

^x^^iv^Peroxisome Proliferator-Activated Receptor Gamma

^x^^v^Basic Fibroblast Growth Factor

^x^^vi^5-Fluorouracil

^x^^vii^Tyrosine Kinase Inhibitor

^x^^viii^Peroxisome Proliferator-Activated Receptor Alpha

^x^^ix^6-Phosphofructo-2-Kinase/Fructose-2,6-Biphosphatase 3

^x^^x^Platelet-Derived Growth Factor Receptor Beta

## Controversial function of stromal cells in cancer

**Fig. 2:**
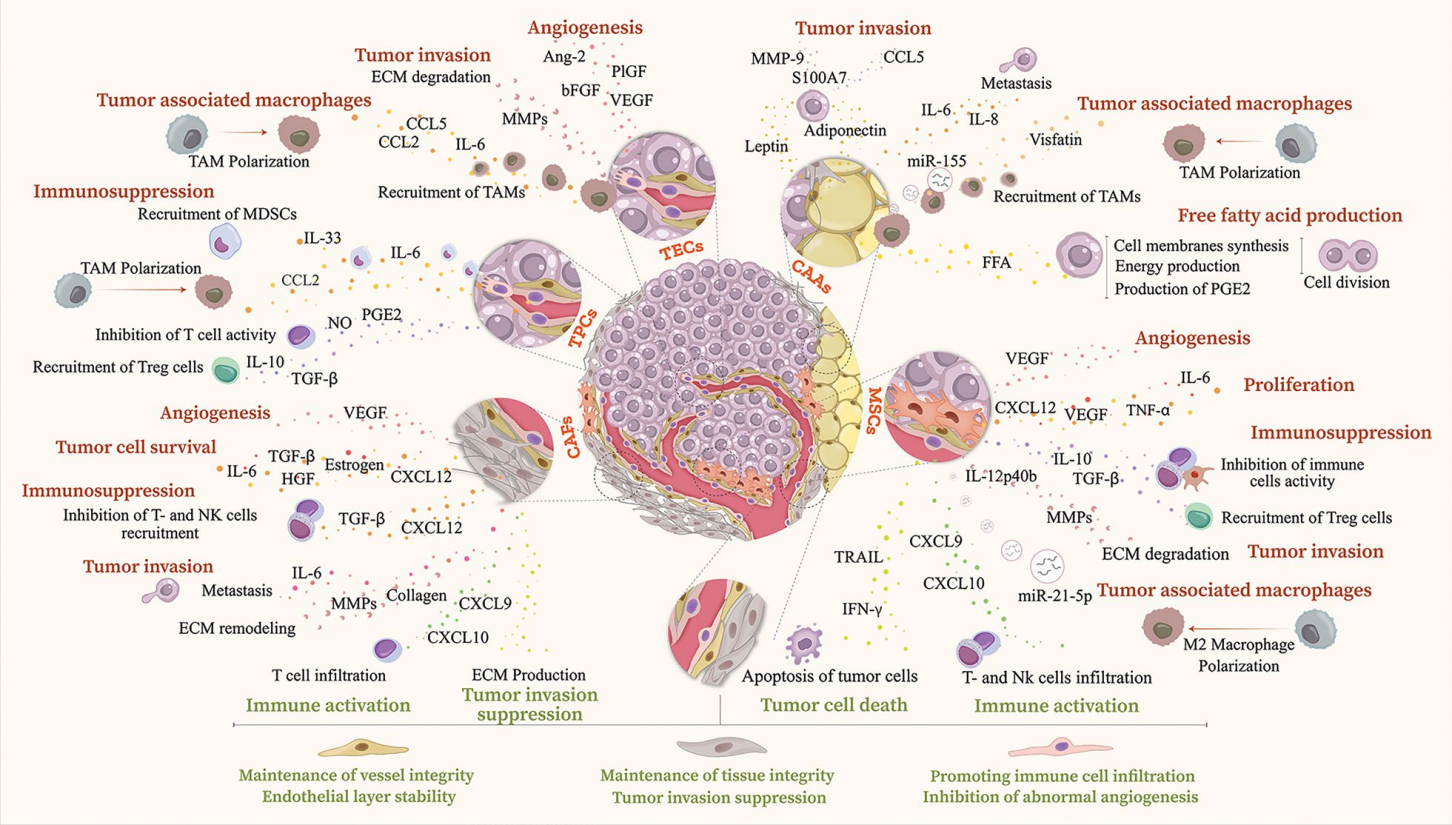
Stromal cells and cancer immunity: a dual role

While stromal cells can augment anti-tumor immune responses, they may also enable immune evasion, consequently advancing tumor growth [10]. In essence, depending on the type of generated factors, the formation of signals, and the interactions among various cell types in the TME, diverse outcomes may arise; ranging from tumor suppression to immune blockade or tumor expansion [175, 176]. This dual nature poses a considerable barrier in formulating therapeutic strategies that utilize stromal cells for cancer treatment [177]. Understanding the mechanisms behind their immunomodulatory functions is crucial for designing strategies that maximize their anti-cancer potential while minimizing pro-tumorigenic effects. The following sections will briefly focus on the controversial function in cancers, attributed to stromal cells. Furthermore, Fig. 2 provides a comprehensive view of the dual role of these cells during cancer progression.

### Stromal cells and cancer progression

The "pro-tumor activity" of stromal cells denotes the capacity of cells in the TME, such as fibroblasts and MSCs, to actively facilitate tumor development, invasion, and metastasis. Stromal cells execute this function through several mechanisms, among which CAFs hold a substantial role [178].

MSCs also produce angiogenic factors, as another tactic for tumorigenic activity [179, 180]. The release of anti-inflammatory factors along with the induction of resistance to treatment are amongst the other schemes of oncogenesis [181]. Accordingly, adipose-derived MSCs promoted the growth of ovarian cancer cells by stabilizing TAZ (transcriptional coactivator with PDZ-binding motif, also known as WWTR1), and this impact seemed to be dependent on MSC-mediated upregulation of the transcription factor PAX8 (Paired-box 8) in ovarian cancer cells [182]. Additionally, MSCs can inhibit anti-tumor immunity by releasing IL-12p40b [76]. Furthermore, the ECM breakdown and matrix remodeling might increase tumor invasiveness due to the expression of MMPs, such as MMP-1, MMP-2, MMP-3, MMP-9, and MMP-13 by human MSCs [76].

Huang et al. demonstrated that colorectal tumor cells secrete elevated levels of endothelin-1 (ET-1), which upregulates angiogenesis through activation of the AKT (protein kinase B) and ERK (extracellular signal-regulated kinase) signaling pathways [183]. Additionally, research indicates that tumor development and metastasis in colorectal cancer are influenced by the activation of the Wnt pathway, primarily through mutations in key regulatory genes such as adenomatous polyposis coli (APC), which is followed by an increase in VEGF expression (184, 185). The impact of vesicles carrying miR-21-5p derived from MSCs on promoting tumor cell proliferation and invasion by reducing apoptosis and promoting macrophage M2 polarization in NSCLS [77], and the vesicles that contain miR-10a originating from bone marrow MSCs in acute myeloid leukemia (AML) was noted in previously conducted research [186]. By promoting the cell cycle entrance and preventing apoptosis, miR-221-3p derived from myeloid leukemia cells increased AML cell proliferation and leukemogenesis [187].

Another study on lung cancer revealed that vesicles containing miR-210-3p and miR-5100 originating from hypoxic BMSCs were helpful in enhancing lung cancer invasion and metastasis [188]. Targeting the epigenetic processes has also been found to be effective in enhancing antitumor immunity in small cell lung cancer [189].

The impact of long non-coding RNAs (lncRNAs) is worth considering in this field. According to the He et al.’s research, gastric tumor cells developed chemo-resistance as a result of MSC-regulated lncRNA-MACC1-AS1’s inhibitory impact on miR-145 [190, 191]. ERK1/2, STAT, and AKT are examples of signaling pathways whose activation contributes to the proliferation of tumor cells [192]. These pathways include the activated p38 mitogen-activated protein kinase (p38MAPK), overactivated JAK-STAT, promoted ERK and AKT, or reduced phosphatase and TENsin homolog (PTEN), acting as tumor suppressors [193–195].

### Anti-cancer activity of stromal cells

Although stromal cells have promoting effect on tumor growth, many investigations have shown that they also possess significant roles in anti-tumor processes [196]. In this regard, these cells seem to hamper tumor cells’ proliferation, progression, and immune evasion by modulating the immune responses in TME [197]. Indeed, the interaction of stromal and immune cells can promote the activity of anti-cancer T cells, such as cytotoxic subtypes, or suppress the activity of regulatory cells, which ultimately both will lead to fighting the tumor. Specific stromal subtypes, such as CAFs, can augment the cytotoxic efficacy of CD8+ T cells and NK cells by releasing chemokines resembling CXCL9 and CXCL10, which promote the recruitment of immune cells to the tumor location [198–201].

Controlling the angiogenesis of tumor cells is another tactic utilized by stromal cells to fight the tumor; this prevents the tumor cells from receiving oxygen and nutrients, which ultimately results in their death [179]. Furthermore, stromal cells have the ability to directly impede the growth of tumor cells by releasing substances, such as cytokines and chemokines, which may cause tumor cells to undergo apoptosis or disrupt signaling pathways that promote tumor development [10, 202].

MSCs have demonstrated the ability of direct induction of cytotoxicity in cancer cells through the secretion of substances, including tumor necrosis factor-related apoptosis-inducing ligand (TRAIL), as well as interferons [203, 204]. These chemicals can trigger apoptosis in neoplastic cells, hence constraining tumor development [180]. Stromal cells can also aid in cancer suppression by participating in ECM remodeling [10]. Normal fibroblasts, unlike their cancer-associated counterparts, preserve tissue integrity and inhibit tumor invasion by strengthening ECM barriers [205]. They can generate enhanced quantities of collagen, along with other structural proteins, including fibronectin, hyaluronan, and laminin, that physically inhibit tumor growth [206]. Enzymes (incl. plasmin, cathepsins, heparanases, and sulfatases) also play a crucial role in the remodeling of ECM [178]. Moreover, MSCs can be designed to transport and deliver anti-cancer medications, such as gemcitabine and paclitaxel to tumors because of their capacity to migrate to tumor locations [207]; this will increase therapeutic efficacy while reducing systemic negative effects [208]. An inhibitory impact on the proliferation of cancer cells was noted in an experiment where bone-marrow-derived MSCs (BM-MSCs) and breast cancer cells (MDA-MB-231) were co-cultured; this brought on the production of TRAIL and ERK1/2 and AKT inhibition, which in turn caused apoptosis, and eventually the suppression of tumor development [209].

In a study involving primary and metastatic B16-F10 melanoma cancer cells in mice, MSCs were employed as IL-12 carriers [210]. It has been demonstrated that IL-12 has anti-cancer efficacy as it triggers a potent immune response against cancer cells. The administration of IL-12 resulted in an increase in the quantity of CD8^+^ cytotoxic T cells and M1-macrophage (producer of pro-inflammatory cytokines), which possess anti-cancer properties, while simultaneously reducing angiogenesis [210, 211]. Moreover, another research has indicated that miR-34a-modified MSCs can cause both glioma cell senescence and DNA damage via the control of Sirtuin 1 (SIRT1) [212].

The decrease in JAK-STAT pathway activity leads to a diminished secretion of cytokines that influence stromal cells, such as IL-6 and IL-10. This reduction subsequently lowers the activity of this pathway within stromal cells and decrease the expression of factors, including NF-KB. Therefore, the decrease in the JAK-STAT signaling pathway activity in tumor cells is one of the elements that can cease tumor development [213]. Moreover, p53 in MSCs can trigger the tumor cells death [214]. A critical prerequisite for the initiation of anti-cancer functions by stromal cells is their targeted homing to the tumor microenvironment, a process mediated by chemokine receptors including CXCR4, CXCR3, CCR4, CCR5, CCR7, and CCR9 [10, 215]. It is worth noting that factors such as tumor type, stromal cell subtype, and the characteristics of the tumor microenvironment substantially influence the anti-tumor efficacy of stromal cells. These complex interactions highlight the pivotal role of stromal cells as integral mediators of immune-driven tumor suppression [142].

## Development and characterization of mature TLSs

**Fig. 3:**
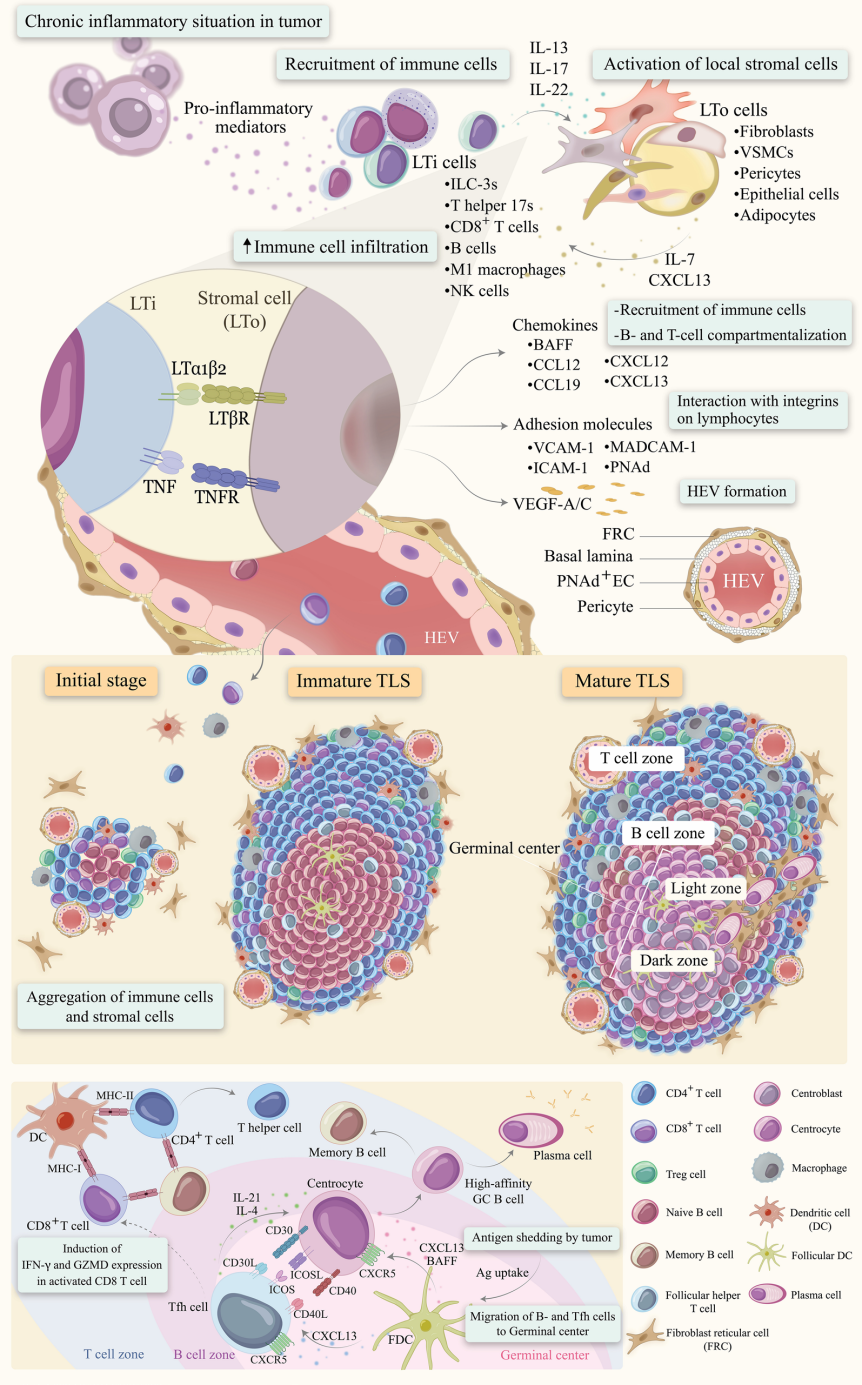
Tertiary lymphoid structures (TLSs): formation, maturation, and characterization

Establishing the lymphoid organs as specialized structures of the immune system, involve in host protection against any harmful situation. In addition to secondary lymphoid organs (SLOs), which are already widely known, TLSs, also termed tertiary lymphoid organs (TLOs) or ectopic lymphoid organs, are organized aggregations of immune and non-immune cells that are linked together by various cytokines and chemokines (Fig. 3). TLSs share similar structure and function with SLOs, but are non-capsulated structures that can form in non-lymphoid tissues under chronic inflammatory conditions such as autoimmune disorders, persistent infections, transplant rejection, and cancers [216–219]. While SLOs are characterized by clear B cell and T cell zones, TLSs are variable in morphology and cellular composition due to different anatomical and disease states, from simple clusters of B and T lymphocytes to well-organized TLSs with B follicles and active germinal centers (GCs) [220, 221]. Various innate and adaptive immune cells such as dendritic cells, macrophages, different subsets of T and B lymphocytes, as well as fibroblastic reticular cells (FRCs), follicular dendritic cells (FDCs), and PNAd + high endothelial venules (HEVs) can be detected in TLSs [222, 223]. TLS formation has been reported in different types of cancers from non-small cell lung (NSCLC), colorectal, and breast cancers, as well as hepatocellular carcinoma (HCC) to pancreatic cancer, ovarian cancer, and melanomas [224–230].

In general, the presence of TLSs at the tumor site are associated with better clinical prognosis, and response to therapies such as ICI therapy can enhance tumor control [231–233]. Effector B and T cells within the TLS, can orchestrate a potent anti-tumor immunity and improve patient prognosis [234]. However, some studies have reported opposite results and adverse effects of TLSs function in colorectal and breast cancers [235, 236].

### Formation and maturation of TLSs

The initiation of SLO development during embryogenesis is orchestrated by interactions between lymphoid tissue organizer (LTo) cells, such as mesenchymal stromal cells, and lymphoid tissue inducer (LTi) cells, mediated through a complex network of cytokines and chemokines [237]. In contrast to SLO formation, studies indicate that chronic inflammation alone can induce the formation of TLSs, even in the absence of classical LTi cells [238]. Within the TME, inflammatory conditions and the accumulation of pro-inflammatory mediators recruit leukocytes that function analogously to LTi cells, leading to the secretion of interleukins IL-7, IL-13, and IL-22. These cytokines activate local stromal cells, which assume the role of LTo cells [220]. Consequently, multiple immune cell types-including innate lymphoid cell type 3 (ILC3), T helper 17 (Th17) cells, CD8+ T cells, B lymphocytes, M1 macrophages, and potentially NK cells-can substitute for LTi cells during TLS induction [239–242].

LTo cells comprise various stromal populations, including fibroblasts, vascular smooth muscle cells, pericytes, epithelial cells, and adipocytes. Notably, fibroblasts can form networks resembling FRCs, which support lymphocyte trafficking within TLSs [243, 244]. The initial stage of TLS formation involves the simple aggregation of immune and stromal cells; however, additional maturation steps are required to establish fully functional TLSs. Activated LTo cells promote further recruitment of LTi-like cells by producing cytokines such as IL-17, CXCL13, and VEGF-A [24, 245]. The interaction between lymphotoxin β receptor (LTβR) and tumor necrosis factor receptor I (TNFRI) on LTo cells with their ligands LTα1β2 and TNF expressed on LTi cells stimulates the secretion of VEGF-C, a critical factor in the formation of high endothelial venules (HEVs) [246]. Kanameishi et al. reported the accumulation of CD11c + dendritic cells (DCs) surrounding nascent HEVs, and depletion of this DC population inhibits HEV formation [247].

LTo cells secrete chemokines including B-cell activating factor (BAFF), CCL12, CCL19, CCL21, CXCL12, and CXCL13, enhancing immune cell infiltration. These chemokines induce lymphocytes to express LTα1β2, promoting the recruitment of T and B cells from HEVs into TLSs, thereby facilitating the establishment of discrete T and B cell zones [248, 249]. TNF and LTα1β2 further induce FDCs, which are essential for B cell zone development [6]. The compartmentalization of T and B cells, orchestrated by GC-specific cytokine profiles-including CXCL12, CXCL13, CCL19, CCL21, and LTα1β2-is indicative of TLS maturation, with the emergence of GCs within follicles representing the final stage of TLS development. T cells expressing CCR7 are recruited by CCL21 and CCL19 to form the T cell zone, whereas CXCR5+/CXCR4+ B cells migrate to follicles via CXCR5/CXCL13 and CXCR4/CXCL12 signaling pathways [250, 251]. Furthermore, it has recently been discovered that the tryptophan-enriched metabolic microenvironment generated by tumor cells involves in the deviation of TLS maturation, while blocking tryptophan metabolism induces intratumoral TLS maturation and enhances tumor control [252].

Within GCs, chemokines CXCL12 and CXCL13 play crucial roles in demarcating the dark and light zones through interacting with their receptors CXCR4 and CXCR5, respectively [253]. Denton et al. demonstrated that following influenza A virus infection in mice, TLS formation occurs, and type I interferon (IFN-I) produced post-infection induces CXCL13 expression in a distinct subset of lung fibroblasts phenotypically different from other stromal populations. CXCL13 recruits CXCR5-expressing B cells into germinal centers, promotes their differentiation into antibody-secreting plasma cells, and facilitates T follicular helper (Tfh) cell development [254]. Gu-Trantien et al. reported that the expression of IL-21, CXCL13, ICOS, and CD200 within TLSs of breast cancer patients signifies the presence of Tfh cells [255]. Consistent with prior knowledge, interactions between Tfh cells and B cells via ICOS-ICOS ligand (ICOSL) and CD40-CD40 ligand (CD40L) pathways are critical for B cell activation, differentiation, and survival [256, 257]. Additionally, Tfh cells enhance the effector functions of activated CD8+ T cells by inducing the expression of IFN-γ and granzyme B (GZMB) [258]. In an ischemia–reperfusion injury (IRI) model of inducible TLS formation in aged mice, interactions between senescence-associated T (SAT) cells-characterized by CD153, PD-1, and CD4 expression and gene profiles resembling human Tfh cells-and B cells via CD153-CD30 signaling are essential for germinal center and TLS expansion [259]. Beyond Tfh cells, fibroblasts contribute to B cell survival through the production of IL-7 and BAFF [244, 260].

Although the mechanisms underlying TLS formation have been extensively investigated in various chronic inflammatory conditions, the precise pathways and mediators involved warrant further elucidation in future studies.

### Characterization of mature TLS

Following the maturation of TLSs, activated immune cells egress from the TLS via HEVs toward the tumor site to potentiate anti-tumor immunity [5]. Recent evidence suggests that only mature TLSs containing GCs are sufficiently effective in mediating tumor control [229].

Mature TLSs are generally characterized by the presence of GCs, which are surrounded by diverse immune cell populations within distinct B and T cell zones, stromal cells, and a network of PNAd + HEVs. The interaction between CXCR4 and CXCR5 receptors on B cells and their respective ligands CXCL12 and CXCL13 facilitates B cell migration into follicles and subsequent GC formation [254, 261]. Although complete segregation of the dark and light zones within GCs is uncommon, such compartmentalization has been documented in the parotid glands of patients with Sjögren’s syndrome [262]. Furthermore, CXCL13 promotes the development of Tfh cells, which localize adjacent to B cell follicles and contribute to B cell differentiation and activation through CD40-CD40L, ICOS-ICOSL, and CD153-CD30 signaling pathways [256, 257, 259].

In addition to BCL6, B cells within GCs express activation-induced cytidine deaminase (AID) and Ki67, markers indicative of affinity maturation, somatic hypermutation, and clonal expansion, reflecting enhanced immune functionality [263]. Germain et al. reported that GC B cells within TLSs of NSCLC patients were positive for Ki67 and BCL6, confirming the presence of mature TLSs in these individuals [264]. CD21 + FDCs, integral to the reticular network, are involved in the selection of memory B cells within GCs and in antigen capture [265]. Moreover, the density of DC-LAMP + dendritic cells in the T cell zone correlates positively with TLS maturation and promotes T cell responses [266, 267].

Studies in lung and ovarian cancers have demonstrated that dendritic cells within TLSs are associated with robust Th1 immune responses and improved prognosis, underscoring the critical role of dendritic cells in antigen presentation and initiation of anti-tumor immunity [228, 268].

### Roles of stroma cells in TLS development

**Fig. 4:**
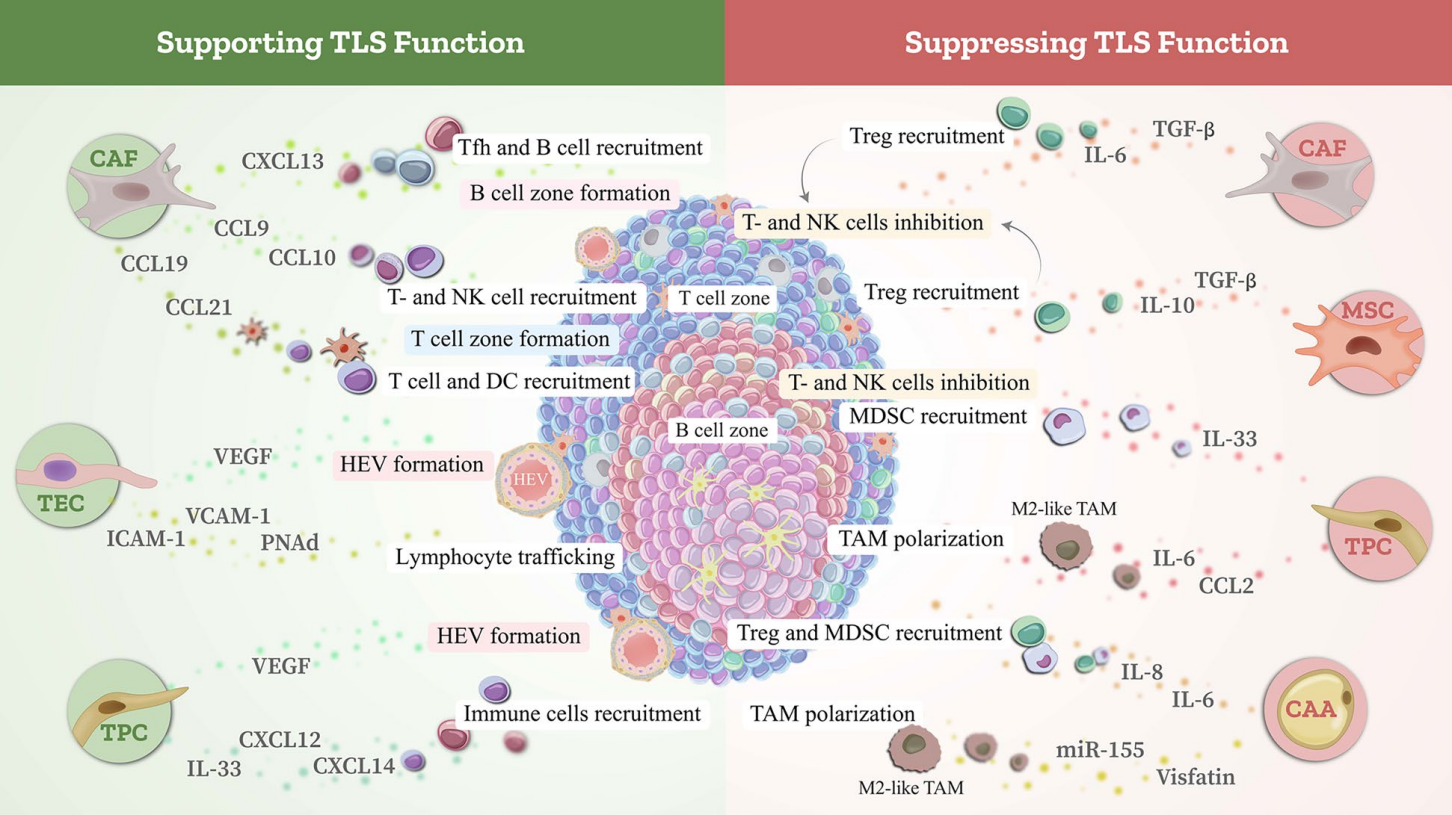
Regulatory interactions among stromal cell subtypes and their role in TLS function

Numerous studies have highlighted the critical supportive roles of specialized stromal cell networks, including fibroblasts, vascular endothelial cells, pericytes, epithelial cells, and adipocytes, in the formation and functional regulation of TLSs [21, 269] (Fig. 4).

Gu-Trantien et al. reported that, in addition to CD4+ T cells, EpCAM + epithelial cells and endothelial/stromal cells expressing markers such as STRO1, endoglin, CD10, or CD271 constitute major sources of CXCL13 production during TLS development [255]. Furthermore, in apolipoprotein E-deficient (ApoE^-/-) mice, activated LTβR-positive aortic SMCs contribute to TLS maturation and GC formation through the secretion of CXCL13, CCL21, and LTβ [270].

Fibroblasts, among the most extensively studied stromal cells in the context of TLS formation, exhibit remarkable plasticity in their functional phenotype; under inflammatory conditions, fibroblasts can acquire LTo-like characteristics, including the secretion of cytokines and chemokines as well as the expression of adhesion molecules, thereby facilitating immune cell recruitment to sites of inflammation and supporting subsequent immune responses [244, 271]. For example, in murine models of inflammatory lesions, fibroblasts phenotypically resemble LTo cells by expressing podoplanin, CXCL13, CCL21, and CCL19, which promotes lymphocyte infiltration into inflamed tissues [272, 273]. Moreover, in both human and murine lung cancer, enhanced CD8+ T cell infiltration and tumor immunity have been correlated with fibroblasts producing CCL19 [274].

Consistent with these findings, Nayar et al. demonstrated that in patients with primary Sjögren’s syndrome (pSS) and in a murine model of induced salivary gland inflammation, a distinct subset of activated fibroblasts-termed immunofibroblasts and characterized by FAP+ and podoplanin + expression-is associated with the early stages of TLS establishment. This process is mediated by IL-13 production, which promotes lymphocyte infiltration and induces the release of IL-22 and LTα1β2 [244].

As previously noted, CXCL13-producing fibroblasts in mice infected with influenza A virus and following IFN treatment enhance B cell differentiation, GC formation, and Tfh cell development [254].

Although numerous studies have documented the positive roles of stromal cells in TLS formation across various inflammatory conditions, MacFawn et al. reported that in high-grade serous ovarian cancer (HGSOC), the TLS-distal stroma undergoes reprogramming toward a pro-tumorigenic phenotype, resulting in the downregulation of TLS-promoting chemokine genes such as CXCL12 and IL-17. Furthermore, they demonstrated that this reprogrammed stroma exhibits impaired adhesion to B cells and fails to differentiate into FDC-like cells in vitro, a finding that contrasts with the requirements for TLS development [275]. This discrepancy may be attributable to factors including the anatomical localization of stromal cells, the nature and severity of the inflammatory milieu, and underscores the need for more comprehensive investigation in this area.

Given the critical involvement of stromal cells in TLS formation and maturation, these cells represent promising targets for novel therapeutic interventions aimed at enhancing localized anti-tumor immune responses. Consequently, the identification and characterization of potential inducers of TLS formation remain areas of considerable interest, albeit with significant challenges to be addressed. For enhanced clarity, Fig. 4 depicts the regulatory interactions between various stromal cell types and the functional dynamics of TLSs.

## Role of TLS in modulating the anti-cancer immunotherapy

**Fig. 5:**
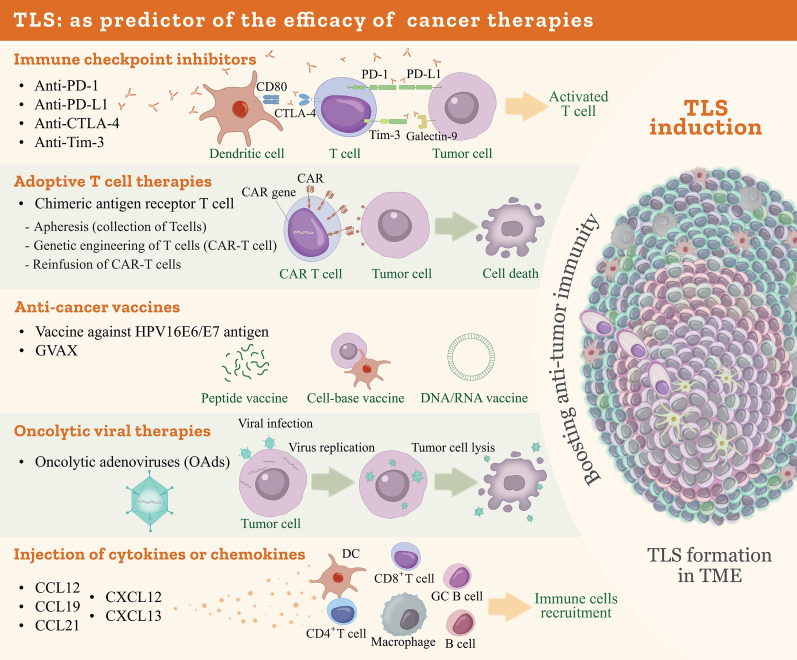
The role of TLSs in modulating anticancer immunotherapy

Immunotherapies, including ICIs, anti-cancer vaccines, CAR-T therapy, and oncolytic viral therapy, have demonstrated the capacity to augment the host’s antitumor immune response. Over recent decades, ICI therapy has emerged as a cornerstone treatment for multiple malignancies and may exhibit enhanced efficacy when combined with adjunctive approaches such as TLS induction [276, 277] (Fig. 5).

Recent studies have revealed that responder patients undergoing ICI treatment exhibit increased infiltration of plasma cells and memory B cells at tumor sites compared to non-responders, reflecting GC activity and TLS formation [222]. In melanoma patients treated with ICIs, the presence of B cells within TLSs correlates with improved prognosis and therapeutic response [227]. Similarly, TLSs and mature B cells have been identified in biopsy specimens from NSCLC patients responding to programmed cell death protein 1 (PD-1) blockade [278]. Moreover, PD-L1 mutations, such as PD-L1^P146R^ have been recognized as prognostic and a negative predictor of response to immunotherapy in cancers, particularly gastric cancer [279].

Eschweiler et al. demonstrated that anti-PD-L1 monotherapy, alone or in combination with anti-CTLA-4 therapy, induces the formation of mature TLSs characterized by distinct light and dark zones within GCs in the B16-OVA mouse melanoma cellular model [280]. Cohort studies of patients with advanced cancers treated with PD-1 or PD-L1 inhibitors have similarly indicated that the presence of mature TLSs-identified by CD3, CD20, and CD23 expression via IHC-predicts favorable immunotherapeutic outcomes [281].

While TLS formation and immune cell aggregation within the TME are predominantly induced by anti-cancer vaccines rather than ICI therapy, evidence supports their critical role in mediating effective antitumor immunity; for instance, in cervical cancer patients, vaccination against the HPV16 E6/E7 antigen promotes the accumulation of T and B cells within the cervical interstitium and induces TLS formation [282]. Moreover, Lutz et al. reported that administration of an irradiated, granulocyte–macrophage colony-stimulating factor (GM-CSF)-secreting allogeneic pancreatic ductal adenocarcinoma (PDAC) vaccine (GVAX), either as a monotherapy or in combination with low-dose cyclophosphamide (Cy) to deplete Tregs, elicits T cell infiltration and TLS development within the TME in over 80% of PDAC patients. Notably, T cell infiltration within TLSs upregulates the PD-1/PD-L1 pathway, suggesting that vaccinated patients may be optimal candidates for subsequent immune checkpoint blockade therapies [283].

Collectively, these findings underscore the potential of TLS induction to enhance antitumor immunity, positioning it as a promising avenue for cancer treatment, particularly in combination with established immunotherapies. Beyond immunotherapy, diverse anticancer modalities-including cytokine or chemokine administration, chemotherapy, radiotherapy, microbial transplantation, and novel biomaterials such as collagen scaffolds, hydrogels, and nanomaterials-have been shown to promote TLS formation. Additionally, advancements in organoid and three-dimensional (3D) culture technologies offer innovative platforms for studying TLS biology and therapeutic induction, as comprehensively reviewed by Zhang et al. [24]. However, the clinical translation of these approaches necessitates careful consideration of safety profiles and the development of reliable humanized in vivo models, warranting further investigation.

### Stroma cells in TLS induction and the role in cancer therapy

Given the therapeutic benefits of TLS induction in cancer treatment, targeting stromal cells-integral components of TLS development-represents a promising strategy to enhance antitumor immunity (Table 2). Cytokines produced by stromal cells, as well as associated signaling pathways, have been implicated as key drivers of TLS formation for therapeutic applications [233].

**Table 2:** Stroma cells in TLSs induction and the role in cancer therapy

Strategy	Treatment	Experimental subjects	TLS detection methods	TLS induction approach	TLS components	References
Cytokines or cytokine-associated signaling pathways	LIGHT-VTP monotherapy and In combination with checkpoint inhibitors of CTLA-4 and/or PD-1	Pancreatic neuroendocrine tumors of RIP1-Tag5 mice	H&E/IHC/RNA analysis	Maturation of pericytes/normalization of blood vessels/increases immune cell infiltration	PNAd+ HEV/FRC/CD11+ DC/Gr1+ or F4/80+ monocyte/CD68+ MQ/Ki67+ GrzB+ CD4+ and CD8+ T cell/distinct T cell and B cell zones	[294]
	LIGHT-VTP monotherapy and in combination with anti-PD-1	Lewis lung carcinoma (LLC) and B16-F10 melanoma (B16) of mice	H&E/Immunofluorescence Staining and Imaging/Flow-cytometery	Enhances expression of pericyte contractile markers/vessel normalization/increases immune cell infiltration	MECA79+ HEV/NK cell/CD11b+ monocyte, macrophag/B cell/CD8+ GrzB+ T cell	[289]
	LIGHT-VTP	Glioblastoma (GBM) of mice	IHC	Inducing pericyte contractility/Normalization of the brain cancer vasculature/increases immune cell infiltration	MECA79+ CD31+ HEV/CD4+ and CD8+ T cell	[300]
	Nano-sapper encoding LIGHT monotherapy and in combination with anti-PD-1	Pancreatic ductal adenocarcinoma (PDAC) of C57BL/6 mice	H&E/IHC/Flow-cytometery	Vessel normalization/increases immune cell infiltration	MECA79+ HEV/CD45+ CD3+ CD8+ T cell/CD45+ CD3+ CD4+ T cell/Treg/CD45+ B220+ B cell/F4/80+ macrophage	[287]
	Photosensitive bacterial system (E@L-P/ICG) loaded with LIGHT	Colorectal cancer of mice	-	generation of HEVs/increases immune cell infiltration	-	[288]
	Brain endothelial cell-targeted adeno-associated viral (AAV) vector expressing LIGHT	Glioma of C57BL/6 mice	IHC/Flow-cytometery/RT-qPCR	Upregulation of HEV-associated genes and activated endothelium/enhances T cell recruitment/promotes a Tstem-like cell phenotype	MAdCAM-1+ CD31+ PNAd+ HEV/CD45+ B220+ B cell/CD45+ CD3+ T cell/Ki67+ proliferating B and T cell/CD11c+ DC/CCL21+ FRCs/T cell zones	[290]
	Scaffolds containing Lα1β2, CCL19, CCL21, CXCL12, CXCL13, sRANKL	Renal subcapsular space of kidneys of mice	IHC	Increase recruitment of immune cells and interaction between LTo cells with LTi cells	HEVs-like structure/B cell and T cell areas/DCs/FDC FRC/CD3^+^CD4^+^CD44^+^CD62L memory T cell/B220^+^CD38^+^IgG^+^ memory B cell	[295]
	Anti-VEGFR2 in combination withanti–PD-L1 antibody	Pancreatic neuroendocrine tumor and breast cancer of mice	IHC/Immunofluorescence staining	Activation of LTβR signaling in tumor vessels/enhances HEV formation/increases T cell infiltration and activation	MECA79+ HEV/CD8+ T cell/CD11+ DC	[301]
	ADU-S@M1 biomimetic nanosystem (M1 cell-derived extracellular vesicles loaded by STING)	mouse fibroblasts (L-929 cells)/CT26 (mouse colon cancer cell line) and B16F10 (murine melanoma cancer cell line) tumor bearing mice	Immunofluorescence staining	increases immune cell infiltration	MECA79+ HEV/CD3+ CD8+ T cell/CD8+ CD44+ T cell/CD8+ CD69+ T cell/CD8+ IFN-ɣ+ T cell/CD19+ B cell/MHC-I+ DC/MHC-I+ M1 cell/	[296]
Transplantation of stromal cells	Modified thymus-derived stromal cell line (TEL-2-LTα)	Renal subcapsular space of kidneys of mice	IHC	TEL-2-LTα acts as LTo cells by expressing LTβR, VCAM-1 and also LTα	MAdCAM1+ HEV/CD11c+ DC/CD4+ and CD8+ T cell/B220+ B cell/CR1+ FDC	[298]
	Lymph node derived stromal cell line	Mice injected with MC38 tumor cells	Flow-cytometery/gene expression and Microarray Assay	Stromal cells act as LTo cells	FRCs/NK cell/macrophage/DC/CD4+ and CD8+ T cell/memory CD4+ and CD8+ T cell (CD44+ CD62L−)	[297]

The cytokine LIGHT (TNFSF14), expressed on activated T cells and immature DCs, interacts with the LTβR expressed on stromal and myeloid cells. LIGHT-LTβR signaling plays a critical role in TLS development by promoting lymphocyte infiltration, vascular normalization, and HEV formation. Recent investigations have focused on the therapeutic induction of LTβR signaling via LIGHT to facilitate TLS generation [284, 285]. It has been demonstrated that intratumoral administration of a recombinant LIGHT RGR peptide in a murine model of pancreatic neuroendocrine tumors induces vascular normalization through the maturation and contractile marker expression of intratumoral pericytes, alongside macrophage activation. Collectively, these effects enhance T and B lymphocyte infiltration and promote the formation of mature TLSs, thereby improving antitumor responses [286].

Delivery of an anti-fibroblastic protein nanoparticle encoding LIGHT stimulates TLS formation by increasing CD8+ T cell recruitment, chemokine production, and tumor vasculature normalization, while concurrently inhibiting aberrant collagen secretion by fibroblasts [287]. Similarly, Hu et al. utilized a photosensitive bacterial system (E@L-P/ICG) loaded with LIGHT cytokine to treat colorectal cancer in mice, resulting in HEV generation, enhanced lymphocyte recruitment, and TLS induction [288]. In another study, He et al. administered a fusion protein comprising LIGHT and a vascular targeting peptide (VTP), which selectively binds tumor vasculature, in murine models of Lewis lung carcinoma (LLC) and B16-F10 melanoma (B16), both representing lung metastasis models. The LIGHT-VTP treatment reduced lung metastases, normalized tumor vasculature, and increased infiltration of CD8+ granzyme B-positive effector T cells. Moreover, combination therapy with LIGHT-VTP and anti-PD-1 immune checkpoint blockade further expanded HEV networks and promoted T and B cell aggregation within metastatic lesions, resembling TLSs. This combinatorial approach significantly enhanced responsiveness to anti-PD-1 therapies in checkpoint inhibitor-resistant lung metastases [289]. Additional preclinical studies have corroborated that LIGHT-mediated therapies and ectopic LIGHT expression in tumors upregulate chemokine and adhesion molecule expression and restore vascular integrity, thereby facilitating TLS development [290–294].

The use of soluble factors secreted by various stromal cells also offers a viable approach for TLS induction. For instance, transplantation of collagen sponge scaffolds impregnated with lymphotoxin-α1β2, CCL19, CCL21, CXCL12, CXCL13, and soluble receptor activator of nuclear factor κB ligand (sRANKL)-all of which are produced by stromal cells-into the renal subcapsular space of mice resulted in the formation of TLSs comprising distinct T and B cell zones, FDCs, FRCs, memory lymphocytes, and HEV structures [295].

Recently, Cheng et al. developed a biomimetic nanosystem (ADU-S@M1) comprising extracellular vesicles derived from M1 macrophages (M1 EVs) loaded with the stimulator of interferon genes (STING) agonist ADU-S100 (ADU-S). This system promotes the polarization of TAMs from the immunosuppressive M2 phenotype to the pro-inflammatory M1 phenotype and facilitates DC maturation. The authors demonstrated that the crosstalk between LTo-like mouse fibroblasts (L-929 cells) and ADU-S@M1–induced M1 macrophages and DCs, functioning as LTi-like cells through secretion of LTα and TNFα, enhances the expression of chemokines CCL19, CCL21, CXCL13, and CXCL20 in L-929 cells. This chemokine upregulation results in effective lymphocyte recruitment and initiation of TLS formation. Supporting these findings, fluorescence imaging of tumor sections from ADU-S@M1–treated CT26 and B16F10 tumor-bearing mice revealed the presence of HEV structures accompanied by clusters predominantly composed of B and T lymphocytes [296].

Alternative approaches for TLS induction include the transplantation of stromal cells; Zhu et al. demonstrated that subcutaneous injection of a lymph node-derived stromal cell line exhibiting fibroblast-like morphology in mice induced TLS formation characterized by the recruitment of diverse immune cell subsets, including B cells, activated CD4+ and CD8+ T cells, DCs, macrophages, and NK cells. Furthermore, they observed that the presence of TLSs inhibited the growth of MC38 murine colon cancer cells in vitro by reducing the expression of immune checkpoint molecules PD-1 and Tim-3 on lymphocytes, thereby enhancing their antitumor activity [297].

Similarly, Suematsu et al. employed a modified thymus-derived stromal cell line, TEL-2-LTα, which expresses LTβR, VCAM-1, and LTα, embedded within a sponge-like collagenous scaffold to engineer lymphoid tissue-like structures. Following transplantation of these stromal cell-laden scaffolds into the renal subcapsular space of mice, infiltration of T and B cells as well as HEV formation were observed, indicative of TLS properties and demonstrating functional immune activation in severely combined immunodeficient (SCID) mice [298].

Advancements in biomaterials have further facilitated TLS induction strategies; for instance, the use of hydrogels incorporating transduced fibroblasts expressing CD40 ligand (CD40L) and B-cell activating factor (BAFF)-designated as the 40LB transgenic cell line-has been shown to promote TLS formation with enhanced GC responses and antibody class switching in vitro, thereby inspiring novel approaches for TLS induction [299].

Collectively, numerous promising strategies have been proposed to enhance cancer immunotherapy through the manipulation of stromal cells to induce TLS formation. These approaches include targeting cytokines or cytokine-associated signaling pathways in stromal cells, such as the administration of LIGHT cytokine via various delivery methods and activation of the STING pathway. Additionally, direct transplantation of stromal cells represents another viable strategy, all of which have demonstrated the capacity to elicit robust immune responses within TLSs. Nevertheless, further comprehensive investigations are warranted to elucidate the precise role of stromal cells in regulating TLS development. The utilization of reliable and physiologically relevant cancer models will be critical to advancing the understanding of stromal cell–TLS interactions and facilitating the development of innovative immunotherapeutic modalities.

## TLSs, as potential biomarkers for cancer prediction and prognosis

As mentioned above, TLSs have garnered increasing interest from both clinicians and researchers as a special component in the TME [302]. A growing body of research shows that TLSs are highly correlated with efficacy and could act as biomarker for predicting and assessing the prognosis of cancer [234, 303]. Despite advances in utilizing ICIs in cancer immunotherapy, predictive markers which are currently used, such as tumor mutational burden and PD-L1/PD-1 expression status, have limitations and encounter challenges in precisely and comprehensively identifying patients with a high likelihood of responding positively to immunotherapy [304]. Research has shown that TLSs may act as potential biomarkers for predicting and monitoring responses to ICI therapy and now they are recognized as a valuable mediator of immunotherapy effectiveness [305, 306].

### The number, density, and location of TLSs affect cancer prediction and prognosis

Although TLSs have been found in several forms of cancer, TLSs exhibit heterogeneity both across different cancer types and among patients and this variety influences patient’s clinical outcome [302]. It is hypothesized that density, location, and maturation stages of TLSs are characteristics that correlate with the positive or negative prognosis of patients [307].

#### TLS location

TLSs can be located in the peritumoral area, invasive margin, and center of tumors [305]. Tumor tissues with intratumoral TLSs demonstrate markedly increased infiltration of T and B cells, accompanied by reduced infiltration of immunosuppressive cells [308]. Thus, it has been reported that intratumoral TLSs are linked with better outcomes for cancer patients including pancreatic cancer and HCC [224, 309]. In contrast, peritumoral TLSs pose a dual role as a prognostic marker. Some studies have reported that in patients with breast cancer, HCC, and colorectal cancer liver metastases, a high level of peritumoral TLSs is associated with less overall survival and worse disease-free survival [310–312]. Whereas in esophageal cancer, the presence of peritumoral TLSs has been linked to lower tumor stages and prolonged survival [313]. Also, TLSs located at the invasive margin have also been validated as significant positive indicators of patient outcomes [314]. According to these findings, different locations of TLSs may either enhance antitumor immunity or contribute to an immunosuppressive tumor environment and the location of TLSs plays a variable role in determining cancer prognosis [305].

##### TLS maturity and density

Maturation of TLSs includes three evolution stages ranging from disorganized cellular clusters containing loose lymphoid aggregates to fully developed primary follicular architectures (consisting of T and B cells with macrophages and DCs) and mature structure (containing GCs, HEVs, and lymphatic vessels) [302, 307]. Infiltration of immune cells, including Tfh cells, proliferating B cells, plasma cells (PCs), and FDCs in high-mature TLS is richer among others enabling optimal interactions among diverse immune cell types causing an effective immune response [304, 315]. While TLSs with low maturity may display a dysfunctional and immunosuppressive phenotype and are associated with poor prognosis in patients [305]. Previous studies have shown that mature TLSs improve the prognosis of ESCC and gastric cancer patients and also supported antitumor adaptive immune responses in PDAC [316–318]; while the presence of low-maturity TLSs was associated with an increased rate of cancer recurrence in HCC and colorectal cancer and also causes tumor invasion, metastasis, and immunosuppression in laryngeal cancer [230, 319, 320]. Thus, mature TLSs have been demonstrated to positively correlate with favorable prognosis and improved immunotherapy responses in cancer patients [307].

The density of TLS differs among individuals, even within the same cancer type and disease stage, highlighting the variability in the TME [321]. Studies have shown that several transcription factors are overexpressed in tumors with high density TLS [221]. Thus, a high density of TLSs is correlated with a better prognosis [307]. According to previous studies, in ovarian cancer, high density of TLS significantly improved overall survival [322].

## TLS detection and quantitative analysis

Research on TLSs within tumor tissues has opened new avenues for the development of innovative immunotherapeutic strategies, underscoring the need for standardized methods for TLS detection and quantification [323]. However, a universally accepted standard for TLS quantification across diverse patient populations and cancer types remains to be established [24]. Current methodologies employed to identify TLSs include hematoxylin and eosin (H&E) staining, multiplex immunohistochemistry (mIHC), multiplex immunofluorescence (mIF), and real-time quantitative polymerase chain reaction (qPCR) targeting chemokine expression profiles [323]. These techniques necessitate the analysis of tumor samples from specific anatomical regions, which limits their applicability, particularly in cases involving inoperable tumors due to insufficient sampling [324].

Consequently, there is a pressing need for indirect and non-invasive approaches, such as the detection of TLS-associated biomarkers, including CXCL13, CCL19/CCL21, CXCL10, and CXCL11, in patient blood or bodily fluids, complemented by advanced imaging modalities. Such strategies are especially valuable for longitudinal monitoring of patients to detect disease recurrence or progression [234, 307, 323]. The advent of artificial intelligence (AI) has further facilitated the automation of image analysis, enabling the potential identification and quantification of TLSs with enhanced accuracy. AI-driven deep learning algorithms have demonstrated considerable promise in early tumor screening, diagnosis, and prognostication across a broad spectrum of malignancies [323]. The integration of specific TLS biomarkers, cutting-edge imaging technologies, and AI analytics holds significant potential to advance TLS detection and quantitative assessment [24, 302]. Nonetheless, this field remains under active investigation. For a comprehensive overview, Table 3 summarizes the most commonly employed TLS detection methods along with their respective characteristics.

**Table 3:** TLS detection strategies

Method of detection	Feature(s)	Reference(s)
H&E staining	Morphology of TLS is detected in tumor sections that are formalin-fixed and embedded in paraffin Low cost with simple execution Unfortunately, Extract limited information. For example, it doesn’t give any information about cellular interactions in the TME	[323]
Multiplex immunofluorescence: mIHC and mIF	Able to conduct a more in-depth investigation into the biology of TME such as intracellular interactions Analysis of multiple markers simultaneously and more accurate in evaluating cell distribution compared to H&E staining	[325]
Genomic signature of TLSs	Detect TLSs quantitatively via evaluating the gene expression of 12 specific chemokines It quantifies and detects TLS at the gene level based on the 12-CK score A high 12-CK score is associated with good prognosis and improved overall survival in patients Only determine the presence of TLSs indirectly	[323, 326]
Flow cytometry	Requires a single-cell suspension of tissue Difficult to detect the presence of TLS due to a homogenous mix of cell population from TLS and non-TLS regions	[327]
3D imaging of TLSs	Non-invasive method Unencapsulated lymphatic aggregates are detected through image scanning Provide information regarding the structure, volume, and number of TLSs and even interactions among the cellular components within TLSs	[323]
AI (Artificial Intelligence)	A valuable tool for automated identification of target cells within tumors Categorization of immune cell subsets according to their morphological features and organizational structure	[323]

## Unresolved issues concerning TLSs in cancer

Despite significant advances in the study of TLSs in recent years, numerous questions and unresolved issues persist. Prior research has demonstrated that TLS formation is a highly orchestrated process involving intricate interactions among hematopoietic cells, non-lymphoid stromal cells, and a variety of molecular mediators. This process is regulated by cytokines, chemokines, adhesion molecules, and survival factors, including CXCL13, CXCL12, CCL19, CCL21, TGF-β, IL-6, and IL-7 [254, 328]. Nevertheless, the precise mechanisms governing TLS development across different pathological contexts, particularly in cancer, remain incompletely understood. Critical questions remain regarding the factors that initiate and sustain TLS formation. Moreover, the reasons underlying the presence of TLSs in only certain cancer types are yet to be elucidated. It is unclear whether specific facilitators or inhibitors govern TLS formation, and the mechanisms driving TLS heterogeneity within a given organ-and the consequent impact on cancer immunity-are still poorly defined [305].

Although TLS presence is generally associated with favorable prognosis, its clinical significance varies depending on disease stage and the cellular composition of TLSs, even within the same malignancy. Furthermore, the influence of TLSs on therapeutic responses, as well as the reciprocal effects of therapy on TLS function, remain inadequately characterized [6]. As discussed, therapeutic induction of TLSs, for example through modulation of stromal cells, represents a promising avenue in cancer treatment. However, despite the potential benefits of enhancing TLS function for tumor control, such interventions may inadvertently provoke autoreactive T and B cell responses in other tissues. This raises the important question of whether TLSs can be therapeutically targeted without compromising systemic immune homeostasis [6, 302].

Several challenges complicate the targeting of stromal cells for TLS manipulation. These include the absence of specific markers that allow selective targeting of TLS-associated stromal cells without disrupting normal stromal architecture in other tissues, the inherent heterogeneity of stromal cells across different organs and disease states, and the complexity of identifying appropriate stromal subpopulations for intervention. Additionally, an incomplete understanding of the precise signaling pathways and molecular cues that induce TLS formation hampers the development of targeted therapies. The delivery of TLS-inducing factors specifically to stromal cells, while avoiding off-target effects, remains technically challenging. Moreover, chronic activation of stromal cells may lead to fibrosis and tissue remodeling, and TLSs themselves may exacerbate autoimmune conditions, rendering their therapeutic manipulation highly context-dependent. Consequently, the potential benefits and risks of strategies aimed at modulating TLSs must be carefully weighed and rigorously evaluated.

## Conclusions and future prospects

The intricate relationship between the TME and cancer progression underscores the importance of understanding the role of TLSs in shaping immune responses. TLSs, which develop in response to chronic inflammation within tumors, serve as critical sites for antigen presentation and lymphocyte activation, potentially enhancing anti-tumor immunity. However, their dual function complicates their role in cancer therapy; while mature TLSs can promote effective immune surveillance, they may also provide niches for immunosuppressive cells that facilitate tumor evasion.

Emerging research highlights the need for a nuanced approach to targeting TLSs in cancer immunotherapy. Strategies aimed at manipulating stromal cells to induce or enhance TLS formation could improve T-cell activation and bolster anti-tumor responses. Nonetheless, the presence of regulatory T cells and myeloid-derived suppressor cells within TLSs necessitates careful consideration of the therapeutic context to avoid exacerbating immune suppression.

Future investigations should focus on elucidating the mechanisms governing TLS maturation and their interactions with various stromal components. Understanding these dynamics will be crucial for developing targeted therapies that optimize the immunogenic potential of TLSs while minimizing their immunosuppressive effects. As advancements in immunotherapy continue to evolve, integrating knowledge about TLSs into therapeutic strategies may significantly enhance treatment outcomes for cancer patients, ultimately leading to more effective and personalized approaches in oncology.

## Data Availability

No datasets were generated or analysed during the current study.

## Abbreviations

ACT: Adoptive cell therapies
AKT: Protein kinase B
AML: Acute myeloid leukemia
APC: Antigen-presenting cell
BAFF: B cell activating factor
BBB: Blood–brain barrier
BM-MSC: Bone-marrow-derived mesenchymal stem cell
CAA: Cancer-associated adipocyte
CAF: Cancer-associated fibroblast
CAR-T: Chimeric antigen receptor T-cell,
CCR-1: C–C chemokine receptor type 1
CDDP: Cisplatin
CDKN1B: Cyclin-dependent kinase inhibitor 1B
CTL: Cytotoxic T lymphocyte
CXCL: Chemokine (C-X-C motif) ligand
Cy: Cyclophosphamide
DC: Dendritic cell
DLL4: Delta-like 4 ligand
ECM: Extracellular matrix
EGF: Epidermal growth Factor
EMT: Epithelial-mesenchymal transition
ERK: Extracellular signal-regulated kinase
FABP: Fatty acid-binding protein
FAP: Fibroblast activation protein
FASN: Fatty acid synthase
FATP1: Fatty acid transport protein 1
FDC: Follicular dendritic cell
FFA: Free fatty acid
FGF: Fibroblast growth factor
FRC: Fibroblastic reticular cell
GC: Germinal center
GM-CSF: Granulocyte–macrophage colony-stimulating factor
GPR77: G-protein-coupled receptor 77
GVHD: Graft-versus-host disease
H&E: Hematoxylin and eosin
HER3: Human epidermal growth factor receptor 3
HEV: High endothelial venule
HGF: Hepatocyte growth factor
HK2: Hexokinase 2
iCAF: Inflammatory CAF
ICI: Immune checkpoint inhibitors
IL: Interleukin
JAK-STAT: Janus kinase/signal transducers and activators of transcription
LncRNA: Long non-coding RNA
LTi: Lymphoid tissue inducer
LTo: Lymphoid tissue organizer
LTβR: Lymphotoxin-β receptor
MACC1: Metastasis-associated in colon cancer 1
MAPK: Mitogen-activated protein kinase
MDSC: Myeloid-derived suppressor cell
mIF: Multiplex immunofluorescence
mIHC: Multiplex immunohistochemistry
MiRNA: MicroRNA
MMP: Matrix metalloproteinase
MSC: Mesenchymal stem cell
myCAF: Myofibroblastic CAF
NEC: Normal endothelial cell
NK: Natural killer cell
NRG1: Neuregulin 1
OAd: Oncolytic adenoviruse
OV: Oncolytic virus
PAX8: Paired-box 8
PC: Pericyte
PDAC: Pancreatic ductal adenocarcinoma
PDGF: Platelet-Derived Growth Factor
PD-L1: Programmed Cell Death Ligand 1
PFKFB3: 6-Phosphofructo-2-kinase/fructose-2,6-bisphosphatase isozyme 3
PGE2: Prostaglandin E2
PPAR-γ: Peroxisome proliferative-activated receptor gamma
PTEN: Phosphatase and TENsin homolog
ROCK2: Rho-associated coiled-coil containing protein kinase 2
SAT: Senescence-associated T cell
SDF-1: Stromal cell-derived factor 1
Shh: Sonic hedgehog signaling
SLO: Secondary lymphoid organ
TPC: Tumor-associated pericyte
TAZ: Transcriptional coactivator with PDZ-binding motif
TEC: Tumor-associated endothelial cell
TFH: T follicular helper cell
TGF-β: Transforming Growth Factor β
TLO: Tertiary lymphoid organ
TLS: Tertiary lymphoid structure
TME: Tumor microenvironment
TNFSF14: Tumour necrosis factor superfamily protein 14
TRAIL: Tumor necrosis factor-related apoptosis-inducing ligand
Treg: Regulatory T cells
TSP-1: Thrombospondin-1
α-SMA: Alpha-smooth muscle actin
